# Dissecting DNA repair in adult high grade gliomas for patient stratification in the post-genomic era

**DOI:** 10.18632/oncotarget.2180

**Published:** 2014-07-09

**Authors:** Christina Perry, Devika Agarwal, Tarek M.A. Abdel-Fatah, Anbarasu Lourdusamy, Richard Grundy, Dorothee T. Auer, David Walker, Ravi Lakhani, Ian S. Scott, Stephen Chan, Graham Ball, Srinivasan Madhusudan

**Affiliations:** ^1^ Academic Unit of Oncology, Division of Cancer and Stem Cells, School of Medicine, University of Nottingham, Nottingham University Hospitals, Nottingham, UK; ^2^ School of Science and Technology, Nottingham Trent University, Clifton Campus, Nottingham, UK; ^3^ Department of Oncology, Nottingham University Hospitals, City Hospital Campus, Nottingham, UK; ^4^ Children's Brain Tumour Research Centre, Division of Cancer and Stem Cells, School of Medicine, University of Nottingham, Queen's Medical Centre, Nottingham University Hospitals, Nottingham, UK; ^5^ Department of Academic Radiology, University of Nottingham, Nottingham University Hospitals, Queen's Medical Centre, Nottingham, UK; ^6^ University of Leicester Medical School, Maurice Shock Building, University Road, Leicester, UK; ^7^ Department of Neuropathology, Nottingham University Hospitals, Queen's Medical Centre, Nottingham, UK

**Keywords:** DNA repair, high grade glioma, glioblastoma, prognostic factor, biomarker

## Abstract

Deregulation of multiple DNA repair pathways may contribute to aggressive biology and therapy resistance in gliomas. We evaluated transcript levels of 157 genes involved in DNA repair in an adult glioblastoma Test set (n=191) and validated in ‘The Cancer Genome Atlas’ (TCGA) cohort (n=508). A DNA repair prognostic index model was generated. Artificial neural network analysis (ANN) was conducted to investigate global gene interactions. Protein expression by immunohistochemistry was conducted in 61 tumours. A fourteen DNA repair gene expression panel was associated with poor survival in Test and TCGA cohorts. A Cox multivariate model revealed *APE1, NBN, PMS2, MGMT* and *PTEN* as independently associated with poor prognosis. A DNA repair prognostic index incorporating *APE1, NBN, PMS2, MGMT* and *PTEN* stratified patients in to three prognostic sub-groups with worsening survival. *APE1, NBN, PMS2, MGMT* and *PTEN* also have predictive significance in patients who received chemotherapy and/or radiotherapy. ANN analysis of *APE1, NBN, PMS2, MGMT* and *PTEN* revealed interactions with genes involved in transcription, hypoxia and metabolic regulation. At the protein level, low APE1 and low PTEN remain associated with poor prognosis. In conclusion, multiple DNA repair pathways operate to influence biology and clinical outcomes in adult high grade gliomas.

## INTRODUCTION

Gliomas are the most common primary central nervous system tumour in adults [1]. Despite advances in surgery, chemotherapy and radiotherapy, patients with grade 3 gliomas (anaplastic astrocytomas, anaplastic oligodendrogliomas and anaplastic oligoastrocytomas) have a five year survival rate of 27% [2]. The outcome for grade 4 gliomas (glioblastoma (GBM)) is even worse [3], with an estimated 2 year survival rate of about 26.5% [3]. Aggressive biology and therapy resistance is a formidable clinical problem. Hence biomarker driven stratification of patients is a high priority.

Alkylating chemotherapeutic agents such as temozolomide, procarbazine and lomustine, as well as radiation therapy, are frequently used in the treatment of high grade gliomas [4, 5]. Although chemotherapy and radiotherapy exert cytotoxic effects through genomic DNA damage, glioma cancer cells, in common with normal cells, have an armoury of DNA repair mechanisms to combat such DNA damage. Proficient DNA repair may promote cancer cell survival leading to treatment resistance and poor clinical outcome. O^6^-methylguanine DNA methyltransferase (*MGMT*) is a key protein involved in the direct repair of O^6^-methylguanine lesions induced by temozolomide chemotherapy. *MGMT* expression status has been extensively studied and has prognostic and predictive significance in gliomas [6]. In a study by Hegi et al, *MGMT* promoter methylation was shown to be present in just under half of all GBM patients. In this same study patients with a methylated *MGMT* promoter who received chemoradiotherapy lived over 6 months longer than those that received radiotherapy alone [7]. However, despite potential *MGMT* status directed therapy most patients will eventually progress and succumb to the disease. This is perhaps not surprising as only approximately 9% of all methyl adducts formed by temozolomide are O^6^-methylguanine lesions and the rest, including N7-methylguanine (the most common, ~70%) and N3-methyladenine, are in fact processed through the DNA base excision repair (BER) machinery in cells [8, 9]. In addition, temozolomide sensitivity may also be influenced by proficient DNA mismatch repair (MMR) in cells [10]. Single strand breaks, generated as DNA repair intermediates during BER or during *MGMT* mediated processing, if unrepaired could eventually lead to accumulation of deleterious double strand DNA breaks (DSBs) [11]. Homologous recombination pathways are required for processing DSBs generated during replication, whereas non-homologous end joining (NHEJ) is essential for the repair of DSBs generated outside the S-phase of the cell cycle [11]. Additional DNA repair pathways, such as nucleotide excision repair (NER) [12] and inter-strand crosslink repair (ICL repair) [13], are also involved in the repair of DNA damaging lesions induced by cytotoxic therapy used in gliomas. DNA repair status may not only predict resistance to therapy but recent emerging evidence also suggests that loss of DNA repair function may lead to accelerated accumulation of mutations during cancer development that eventually drive a mutator phenotype characterised by aggressive biological behaviour and adverse outcomes in patients [14].

Our hypothesis is that, besides MGMT deregulation, multiple other DNA repair pathways may contribute to aggressive biology and poor outcomes. In the current study, we have comprehensively evaluated the transcript levels of 157 genes known to be involved in multiple DNA repair pathways in a Test dataset of 191 tumours and then validated in ‘The Cancer Genome Atlas’ dataset comprising 508 tumours. The data presented here provides evidence that multiple DNA repair pathways operate together to influence outcomes in high grade gliomas.

## RESULTS

### DNA repair gene expression and survival in adult glioblastomas

Univariate associations between expression of 157 DNA repair genes and survival, in the Test set as well as in the TCGA dataset was conducted and followed by Benjamini and Hochberg False Discovery Rate calculation (BH FDR) correction. After applying the BH FDR correction 14 probes (for 12 genes) remained significantly associated with survival in both datasets (Table 1).

**Table 1:** DNA repair genes associated with poor survival in the Test and the TCGA datasets

Gene	Probe	Level associated with worse survival	*P* value (Test dataset)	*P* value (TCGA dataset)
*APE1*	210027_s_at	Low	0.003	0.000018
*PARP2*	204752_x_at	Low	0.014	0.014
*ERCC6*	207347_at	Low	0.006	0.010
*RAD21*	200607_s_at	Low	0.001	0.004
*PTEN*	204054_at	Low	0.004	0.001
*NBN^1^*	202905_x_at	Low	0.000001	0.001
*MGMT*	204880_at	High	0.003	0.001
*BRCA1*	214727_at	High	0.009	0.002
*PMS2*	209805_at	High	0.007	0.011
*PARP3*	209940_at	High	0.002	0.004
*DDB2*	203409_at	High	0.000097	0.00005
*RAD23B*	201222_s_at	High	0.000254	0.006

1 Most significant of 3 probes for NBN shown. p values less than or equal to 0.05 are significant.

### *APE1, NBN, PMS2, MGMT* and *PTEN* mRNA expression levels independently associated with poor prognosis in adult glioblastomas

**Figure 1:**
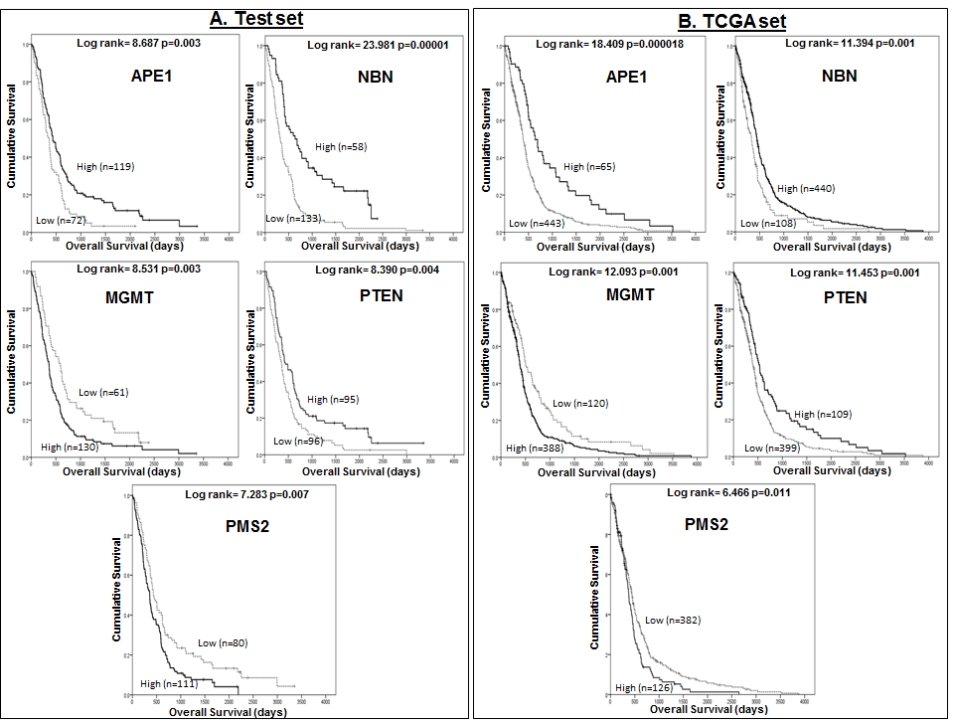
A Kaplan Meier survival curves for overall survival in glioblastoma patients in the Test (A) and TCGA (B) datasets stratified by mRNA expression of *APE1, NBN, MGMT, PTEN*, and *PMS2*.

On multivariate cox regression analysis in the Test dataset, *APE1* (p=0.000810), *Rad23B* (p=0.000167), *PMS2* (p=0.000190), *NBN* (p=0.000846), *MGMT* (p=0.001326) and *PTEN* (p=0.001108) were independent predictors of survival in GBM. In the TCGA dataset, *APE1* (p=0.000128), *PMS2* (p=0.012998), *NBN* [202905_x_at p=0.000025 and 202907_s_at p=0.003634), *BRCA2* (p=0.000188), *MGMT* (p=0.002090) and *PTEN* (p=0.001221) were independently associated with survival. As *Rad23B* (Test dataset), *NBN* [202907_s_at] and *BRCA2* (TCGA dataset) were only significant in one of the datasets, we excluded these probes and repeated the multivariate analyses. The final multivariate models including *APE1, MGMT, NBN, PMS2* and *PTEN* in both datasets are shown in Table 2. Kaplan Meier survival curves for *APE1, NBN, PTEN, PMS2* and *MGMT* in the Test and TCGA datasets are shown in Figure 1.

**Table 2:** Multivariate analysis in the Test and TCGA datasets

Gene	Test dataset		TCGA dataset	
	HR (95% CI)	*P* value	HR (95% CI)	*P* value
*APE1* (210027_s_at)	0.62 (0.45-0.85)	0.002616	0.57 (0.42-0.77)	0.000311
*PMS2* (209805_at)	1.84 (1.32-2.55)	0.000299	1.47 (1.17-1.86)	0.000970
*NBN* (202905_x_at)	0.48 (0.34-0.69)	0.000053	0.60 (0.48-0.77)	0.000045
*MGMT* (204880_at)	1.55 (1.09-2.19)	0.013562	1.41 (1.11-1.79)	0.004329
*PTEN* (204054_at)	0.62 (0.45-0.84)	0.001994	0.67 (0.52-0.86)	0.001575

p values less than or equal to 0.05 are significant.

### DNA repair prognostic index in adult glioblastomas:

We then developed a prognostic index (see methods section) incorporating *APE1, NBN, PTEN, PMS2* and *MGMT*. As described in the methods section, we initially calculated prognostic indices separately for the Test data dataset (PI_1) and the TCGA dataset (PI_2). A combined prognostic index (PI_3) was then generated using the mean β value for each gene from the two datasets.

PI_3 can be described by the formula:

PI_3 = (APE1*−0.524) + (PMS2*0.498) − (NBN*0.620) + (MGMT*0.391) − (PTEN*0.439)

**Figure 2:**
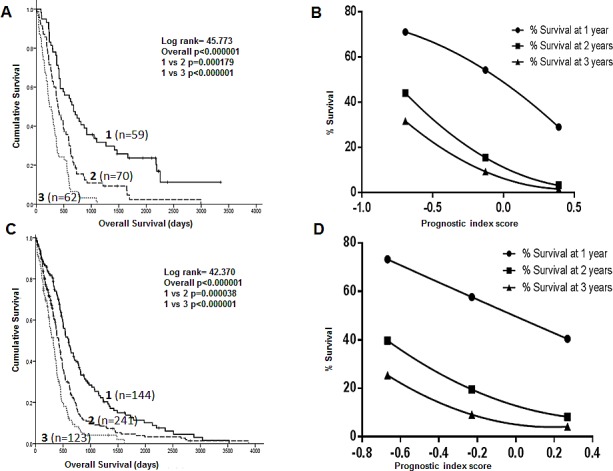
Kaplan Meier survival curves showing separation of patients into 3 prognostic groups by a DNA repair gene prognostic score in the Test dataset (A) and the TCGA dataset (C) Survival curves using the DNA repair gene prognostic index score to predict 1, 2 and 3 year survival in the Test (B) and TCGA (D) datasets.

The PI_3 can separate patients with GBM into three prognostic sub-groups in both the Test set (Figure 2A) and TCGA set (Figure 2C). Patients in prognostic group 1 have a significantly better prognosis than patients in prognostic group 3 (p_1_<0.000001, p_2_<0.000001), where p_1_ is the p value in the Test dataset and p_2_ is the p value in the TCGA dataset.

Using PI_3, curves were constructed to predict 1, 2 and 3 year survival in GBM patients in both the Test and TCGA datasets. Firstly, Kaplan Meier survival life tables were analysed to determine the percentage of patients alive at 1, 2 and 3 years. The percentage survival at 1 year (y axis) was plotted against the median prognostic score for patients within each of the 3 prognostic groups (x axis) and a 2^nd^ order polynomial curve fitted to the data. This process was repeated for 2 and 3 year survival. As shown in Figures 2B (Test set) and 2D (TCGA set), the prognostic index score can be used to predict survival at 1, 2 and 3 years for individual patients. For example, patients with a prognostic index score of −0.1 have a 15-20% chance of surviving to 2 years based on the curves shown in Figures 2B and 2D. The equations for the predictive curves are shown in Supplementary Table S1.

### Predictive significance of *APE1, NBN, PMS2, MGMT* and *PTEN* mRNA expression in adult high grade glioma

**Figure 3:**
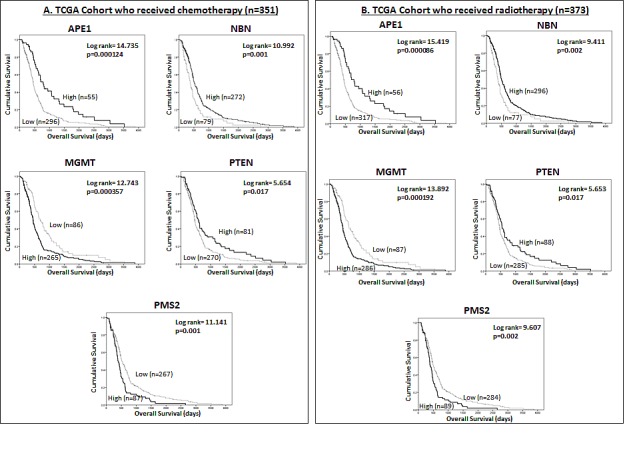
Kaplan Meier survival curves for overall survival in the TCGA dataset for glioblastoma patients treated with chemotherapy (A) and radiotherapy (B) stratified by mRNA expression of *APE1, NBN, MGMT, PTEN*, and *PMS2*.

We have demonstrated that *APE1, NBN, PMS2, MGMT* and *PTEN* have prognostic significance. To investigate whether these DNA repair genes are predictive markers of response to treatment we performed an exploratory sub-group analysis in the TCGA dataset. Kaplan Meier survival analysis was performed separately in patients who had received chemotherapy, and then in those had not received chemotherapy, during the course of their illness. The same methods were applied to patients who had, and had not, received radiotherapy. In patients that received chemotherapy during the course of their illness low *APE1* (p=0.000124), low *NBN* (p=0.001), high *PMS2* (p=0.001), high *MGMT* (p=0.000357) and low PTEN (p=0.017) mRNA expression were associated with poor survival (Figure 3A). In patients who did not receive chemotherapy, only *NBN* (p=0.005) and *PTEN* (p=0.025) mRNA expression were significantly associated with overall survival (Supplementary Figure 1A). Similarly in patients that received radiotherapy low *APE1* (p=0.000086), low *NBN* (p=0.002), high *PMS2* (p=0.002), high *MGMT* (p=0.000197) and low PTEN (p=0.017) mRNA expression were associated with poor survival (Figure 3B) while in patients who did not receive chemotherapy only *NBN* (p-=0.048) mRNA expression was significantly associated with overall survival (Supplementary Figure 1B).

### *APE1, PMS2* and *PTEN* mRNA expression levels are associated with age in adult glioblastomas

To investigate whether age may influence abnormal DNA repair gene expression, and affect susceptibility to the development of gliomas, we assessed the association between *APE1, NBN, PMS2, MGMT* and *PTEN mRNA* expression and age. Low *APE1* (p<0.001), low *PTEN* (p<0.001) and high *PMS2* (p=0.016) were associated with increasing age at diagnosis. No significant associations were seen between *NBN* and *MGMT* expression and age (Supplementary Table 2).

### *APE1, NBN, PMS2, MGMT* and *PTEN* mRNA expression in paediatric high grade gliomas

The data presented in adult tumours provide compelling evidence for the role of *APE1, NBN, PMS2, MGMT* and *PTEN* in gliomagenesis. Moreover, we also observed an age related dysregulation of *APE1, PMS2* and *PTEN* in adult tumours. To evaluate whether *APE1, NBN, PMS2, MGMT* and *PTEN mRNA* also have a role in paediatric high grade gliomas we proceeded to investigate mRNA expression in 53 paediatric high grade gliomas and 27 paediatric glioblastomas. Patient demographics are shown in supplementary Table 3. As summarized in supplementary Table 4, there were no significant associations detected between *APE1, NBN, PMS2, MGMT, PTEN* mRNA expression levels and survival in both datasets after the Bonferroni correction for multiple testing of 16 probesets. Kaplan Meier survival analysis with the dichotomized expression levels (low: < median; high: > median) also revealed non-significant associations between DNA-repair genes and survival in paediatric high-grade gliomas (Supplementary Figure 2). Together the data implies that *APE1, NBN, PMS2, MGMT and PTEN* do not influence paediatric glioma pathogenesis.

### Artificial neural network analysis in adult glioblastomas

The DNA repair association data presented here suggest that *APE1, NBN, PTEN, PMS2* and *MGMT* together may contribute to aggressive biology and influence outcome in patients. An ANNs modelling based, data mining approach was used to identify the gene probes best able to predict expression of selected DNA repair genes (*APE1, NBN, PTEN, PMS2* and *MGMT)*. All ANN analysis was performed in the TCGA dataset (n=508). The algorithm prevented over-fitting of the data by incorporating a constrained architecture and a 3 way Monte Carlo cross validation. Each of the 22,277 probes were utilised singly. The association of each probe with *APE1, NBN, PTEN, PMS2* and *MGMT* expression was assessed based on the model performance. Probes were ranked on their ability to predict *APE1, NBN, PTEN, PMS2* or *MGMT* expression based on the root mean squared error of the model. This technique has been described previously by Lancashire et al [15]. Subsequently, the top 200 probes able to predict expression of our DNA repair genes of interest were selected and a further ANN based network inference algorithm applied [16] which identifies the pairwise interactions between these probes. This method calculates a magnitude and direction of the interaction of each potential pair of probes, a total of 39,800 possible interactions ((200 x 200) − 200).

**Figure 4:**
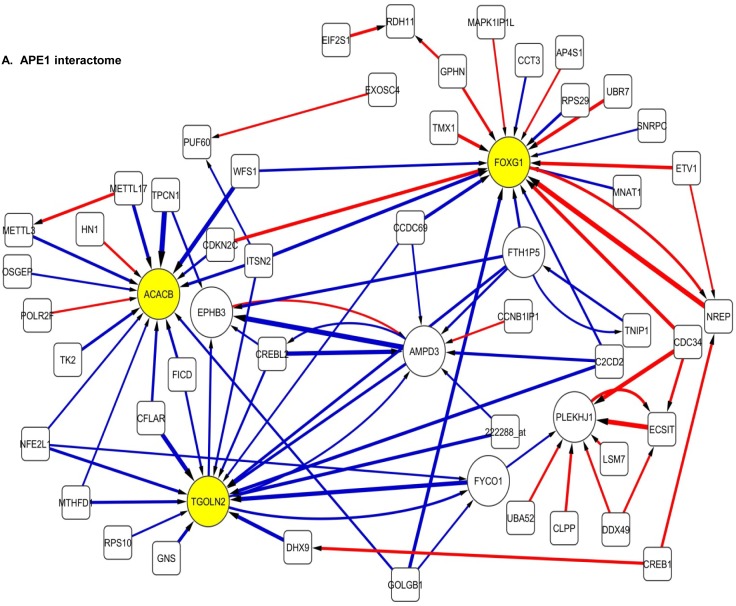

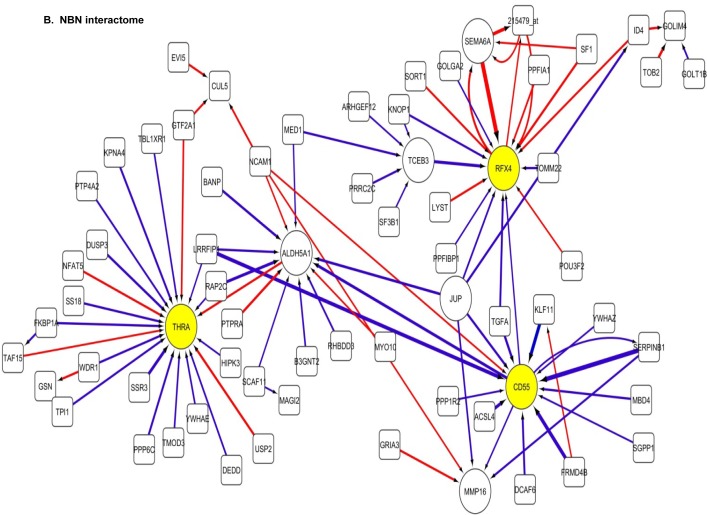

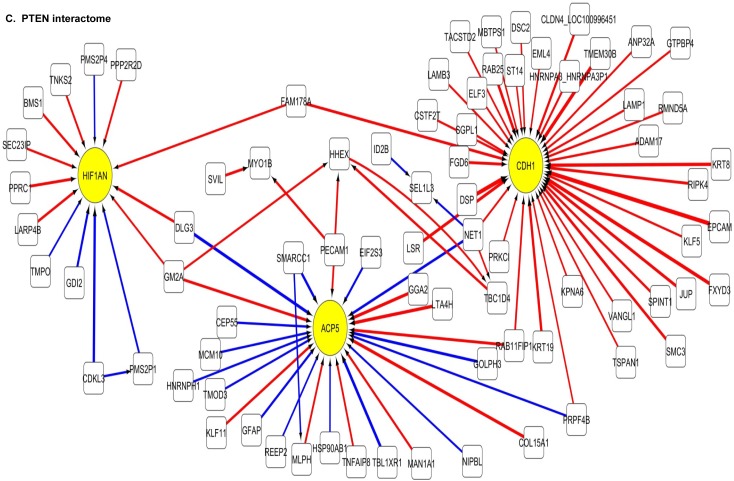

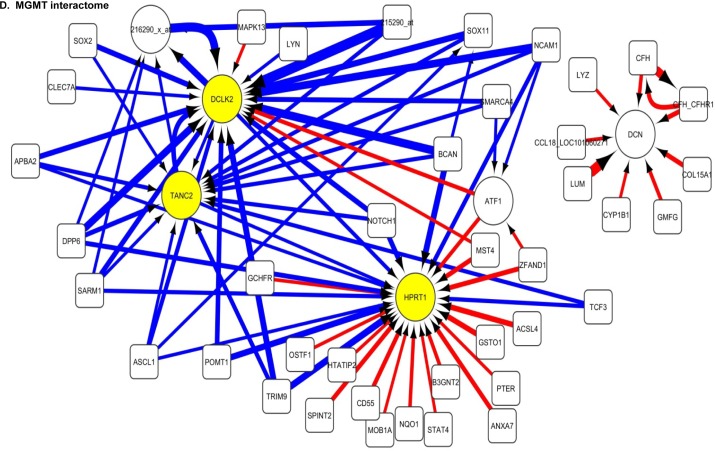

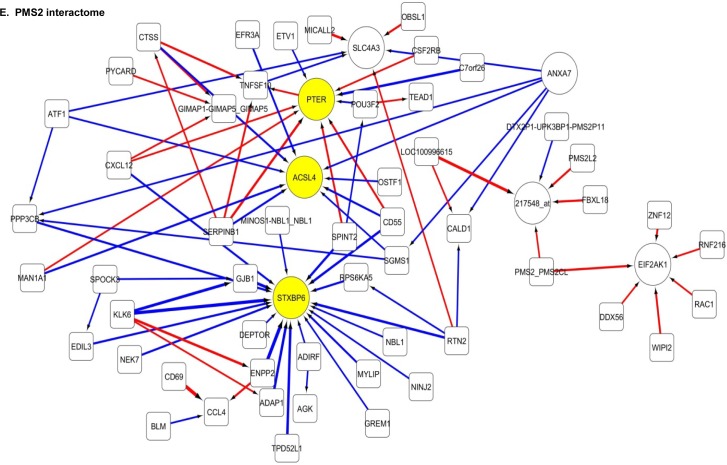
Artificial neural network analysis in TCGA dataset Top pair-wise interactions for gene probe markers associated with *APE1, NBN, PTEN, MGMT* and *PMS2* expression are shown here. Each gene probe is represented by a node and the interaction weight between them as an edge, the width being defined by the magnitude of the weight. Interactions are directed from a source gene to a target gene as indicated by arrows. Red interactions indicate an excitatory interaction and blue indicates an inhibitory interaction. Highly linked genes represent hubs that are likely to be highly influential or highly regulated in the *APE1, NBN, PTEN, MGMT* and *PMS2* systems. Interactome diagrams to show the top 100 interactions of *APE1, NBN, PTEN, MGMT* and *PMS2* are shown here. See results section and supplementary Tables S5-S9 for functions of individual genes.

The 100 interactions having the highest weighting were selected and visualised for *APE1, PMS2, NBN, MGMT* and *PTEN* in a network map (Figure 4). The functions of genes involved in each of the five networks are shown in Supplementary tables S5 to S9 respectively. Key hubs (defined as probes having a large number of interactions (≥5) with other probes either targeting the node or being targeted by the node) can be seen in all five networks and are represented by large circles. It is likely such hubs will have the most influence on the system. The three highest ranked hubs within each network, based on the strength of their interactions, are shaded yellow. The magnitude of the interaction is represented by the width of the line, with positive interactions shown as red arrows and negative interactions as blue arrows. The *APE1* interactome shows *FOXG1, TGOLN2* and *ACACB* as hubs; the *PMS2* interactome shows *STXBP6, PTER* and *ACSL4* as hubs; the *NBN* interactome shows *THRA, RFX*4 and CD55 as hubs; the *MGMT* interactome shows *HPRT, DCLK2* and *TANC2* as hubs; and lastly the *PTEN* interactome shows *CDH1, ACP5* and *HIF1AN* as hubs.

The clinical relevance of these hubs was further investigated in a Cox multivariate model which also included *APE1, PMS2, NBN, MGMT* and *PTEN*. The data is summarised in Table 3. *FOXG1, TOGLN2, DCLK2, THRA, RFX*4, *STXBP6* and *HPRT1* remain independently associated with poor survival. Of note *APE1, PMS2, NBN*, MGMT and PTEN remained independently significant in this analysis.

**Table 3:** Multivariate analysis of *APE1, MGMT, NBN, PMS2, PTEN* and the top 15 hubs identified from artificial neural network analysis

Gene	HR (95% CI)	*P* value
*APE1*	1.888 (1.361-2.619)	0.000141
*MGMT*	0.650 (0.503-0.838)	0.001
*NBN*	1.757 (1.317-2.344)	0.000128
*PMS2*	0.726 (0.572-0.922)	0.009
*PTEN*	1.573 (1.206-2.051)	0.001
*FOXG1*	0.560 (0.449-0.699)	<0.000001
*TOGLN2*	0.715 (0.546-0.937)	0.015
*DCLK2*	0.726 (0.557-0.945)	0.017
*THRA*	1.252 (1.006-1.558)	0.044
*RFX4*	1.445 (1.091-1.913)	0.010
*STXBP6*	0.739 (0.580-0.942)	0.014
*HPRT1*	0.752 (0.584-0.968)	0.027
*TANC2*	0.920 (0.680-1.245)	0.590
*CD55*	0.841 (0.611-1.157)	0.286
*ACACB*	0.819 (0.607-1.106)	0.192
*ACP5*	1.051 (0.800-1.381)	0.720
*HIF1AN*	1.228 (0.976-1.546)	0.079
*ACSL4*	1.242 (0.978-1.578)	0.076
*PTER*	1.247 (0.882-1.762)	0.211
*CDH1*	0.909 (0.695-1.188)	0.485

Significant p values (≤0.05) are shown in bold.

### *APE1, NBN, PMS2, MGMT* and *PTEN* protein expression in adult high grade gliomas

**Figure 5:**
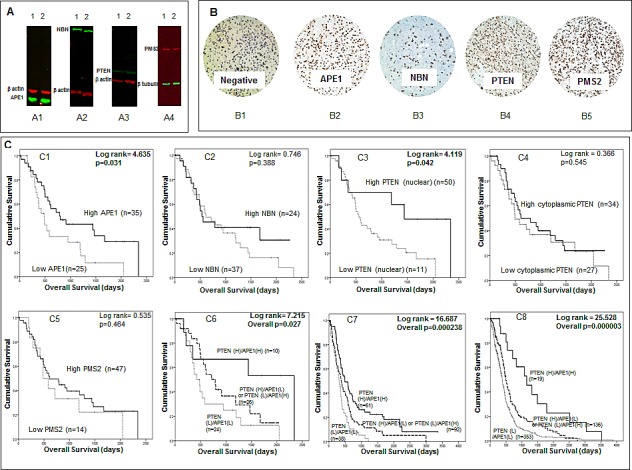
A Western blots demonstrate expression of *APE1* (A1), *NBN* (A2), *PTEN* (A3) and *PMS2* (A4) in LN229 (1) and LN18 (2) glioma cell lines. B Human glioma sections stained using immunohistochemistry technique and the addition of no primary antibody (B1), *APE1* (B2), *NBN* (B3), *PTEN* (B4) and *PMS2* (B5). C Kaplan Meier survival curves for overall survival in high grade glioma patients in the Nottingham cohort stratified by *APE1* (C1), *NBN* (C2), nuclear *PTEN* (C3), cytoplasmic *PTEN* (C4), *PMS2* (C5) and *APE1/PTEN* combination (C6) protein expression. Also Kaplan Meier survival curves for overall survival in glioblastoma patients in the Test (C7) and TCGA (C8) datasets stratified by *APE1/PTEN* combination mRNA expression.

The data presented above provides evidence that *APE1, NBN, PMS2, MGMT* and *PTEN* mRNA expression levels have prognostic and predictive significance in adult tumours. ANN analysis suggests that these genes also interact with genes involved in transcription, hypoxia and metabolic regulation. To investigate whether the prognostic and predictive significance also operates at the protein level, we evaluated a cohort of 61 adult high grade glioma patients treated at Nottingham University Hospitals. As the prognostic significance of low MGMT protein expression has been extensive investigated previously [7], we focussed on APE1, NBN, PMS2 and PTEN protein expression. To evaluate the suitability of the antibody used here, we first investigated the protein expression of APE1, NBN, PMS2, and PTEN in LN229 and LN18 human glioma cell lines by Western blot analysis. As shown in Figure 5A, robust expression of APE1, NBN, PMS2, and PTEN was evident in both cell lines. We then proceeded to immunohistochemical investigations. Clinicopathological association of APE1, NBN, PMS2, and PTEN expression is summarized in Supplementary tables S10 to S14 respectively. APE1 nuclear staining was observed in tumours of all patients (Figure 5B2). Median APE1 H-score was 170 (range 50-250). Low APE1 expression was associated with low PTEN expression (p=0.037). On Kaplan Meier survival analysis, low APE1 (p=0.031) was significantly associated with poor overall survival (Figure 5C1). Nuclear NBN expression was observed in tumours of all patients (Figure 5B3); two tumours also showed cytoplasmic staining. Median NBN expression was 70 (range 0-250). There were no clinicopathological associations with NBN. No significant association (p=0.388) with survival was observed in tumours with low or high NBN expression (Figure 5C2). For PTEN expression, both nuclear (Figure 5B4) and cytoplasmic PTEN staining was seen in the cohort. Median H-score for nuclear PTEN staining was 10 (range 0-105) and median H-score for cytoplasmic PTEN expression was 50 (range 0-200). Thirteen patients were negative for both nuclear and cytoplasmic PTEN staining. Low nuclear PTEN expression was associated with grade 4 gliomas (p=0.006). Similarly, low cytoplasmic expression was also associated with grade 4 gliomas (p=0.034). On Kaplan Meier survival analysis, low nuclear PTEN (p=0.042) expression was significantly associated with poor overall survival (Figure 5C3). Cytoplasmic PTEN expression was not associated with survival (p=0.545, Figure 5C4). Nuclear PMS2 expression was seen in all tumours and the median expression H score was 155 (range 20-250) (Figure 5B5). High PMS2 was associated with grade 4 tumours (p=0.010). No significant association (p=0.464) with survival was observed in tumours with low or high PMS2 expression (Figure 5C5). Comparing APE1, PTEN, NBN, and PMS2 expression in grade 2, 3 and 4 tumours only PTEN expression was associated grade, with increasing grade being associated with increasing levels of PTEN expression (p=0.004). Neither APE1, NBN, PMS2, nor PTEN protein expression levels were associated with age (Supplementary table 2). We also explored whether APE1, NBN, PMS2 and PTEN protein expression have predictive significance in this small cohort. Although APE1, NBN, PMS2 and PTEN protein expression were not associated with survival in patients treated with radiotherapy, low PTEN protein expression was significantly associated with poor survival (p=0.023) in patients treated with chemotherapy (data not shown).

The data presented above suggest that APE1 and PTEN protein expression may have prognostic significance. Interestingly, a recent preclinical study suggested a functional link between APE1 and PTEN [17]. APE1 was shown to transcriptionally regulate PTEN expression [17]. We therefore analysed APE1 and PTEN together in an exploratory study (Figure 5C6). Patients with tumours that had low expression of PTEN and APE1 (n=24) had the worst survival compared tumours that had high expression of PTEN and APE1 (n=10) (p=0.027). Tumours that were PTEN (low)/APE1 (high) or PTEN (high)/APE1 (low) (n=26) had intermediate prognosis (Figure 5C6). To validate whether such a relationship also exists at the mRNA level we investigated *APE1* and *PTEN* together in the Test and the TCGA cohorts. As shown in Figures 5C7 and 5C8, patients with tumours that had low mRNA expression of *PTEN* and *APE1* had the worst survival compared to tumours that had high mRNA expression of *PTEN* and *APE1* in Test set (p=0.000238) as well as in the TCGA cohort (p=0.000003). Taken together, the data provides evidence that *APE1* and *PTEN* have prognostic significance in high grade gliomas.

## DISCUSSION

This is the first study to comprehensively investigate DNA repair in high grade gliomas in the post-genomic era. We have shown that besides *MGMT*, a well-established prognostic and predictive biomarker [7], *APE1, NBN, PMS2* and nuclear *PTEN* may also independently influence survival. Whereas *MGMT* is involved in direct repair [18], *APE1* is critical for base excision repair (BER) [19], *NBN* is a component of the *MRE11*-*RAD50*-*NBN* (MRN) complex involved in DNA damage signalling [20], *PMS2* is essential for mismatch repair (MMR) [21] and recent evidence suggests that nuclear *PTEN* has an important role in DNA double strand break repair and genomic stability [22-24]. In the current study, in univariate as well as in multivariate analysis, low *APE1* mRNA, low *NBN* mRNA and low *PTEN* mRNA levels were associated with poor survival. On the other hand, high *MGMT* mRNA and high *PMS2* mRNA expression were associated with poor survival. Interestingly in paediatric tumours we did not observe any significant associations, implying that the pathogenesis in paediatric tumours is unrelated to the function of these markers. Taken together, the data suggest a complex interaction across multiple DNA repair pathways influencing gliomagenesis in adults but not in children.

Whereas, the favourable prognostic and predictive significance of *MGMT* silencing through promoter hypermethylation has been well established [7], the association between high *PMS2* mRNA and poor prognosis was interesting. Germ-line mutation and loss of *PMS2* is associated with Turcot's syndrome (TS), a variant of hereditary non-polyposis colorectal cancer (HNPCC) syndrome, characterised by colonic polyposis and brain tumours. In a review of 100 cases of Turcot's syndrome, TS patients with glioblastoma survived longer than patients with sporadic glioblastomas [25, 26]. The data presented here, demonstrating that low *PMS2* mRNA is associated with improved survival in adult glioblastoma patients, would concur with the improved survival seen in TS patients. However it is important to note that PMS2 expression in sporadic paediatric tumours did not influence outcome in the current study. Previous studies have described a hypermutated phenotype in glioblastoma patients with mutations in MMR genes. In one such study, 6 out of 7 hypermutated tumours had mutations in one of the MMR genes *MLH1, MSH2, MSH6* and *PMS2* compared to only one tumour in 84 non-hypermutated tumours [27]. This may explain why in our study adult patients with low *PMS2* expression were younger at the time of GBM diagnosis. We speculate that *PMS2* may have essential roles in adult glioma pathogenesis but detailed mechanistic studies are required to confirm this hypothesis.

Another unexpected finding in the current study was that low *APE1* was associated with poor survival. The prognostic significance of low *APE1* was demonstrated at the mRNA and protein level. This is in contrast to a previous study by Bobola et. al., where high AP endonuclease activity was observed in high grade gliomas when compared to low grade gliomas or normal brain tissue [28]. Although there was evidence of increased *APE1* by Western blots in paired samples, the study did not investigate *APE1* mRNA expression or *APE1* protein expression by immunohistochemistry or correlate to survival. An additional limitation of that study was that it included only 39 glioblastomas samples [28]. Although high *APE1* has been demonstrated in multiple tumour types and associated with poor prognosis or response to therapy [9, 29], in a recent study in a large cohort of breast cancers (n=1285), we observed that *APE1* was low in about 50% of tumours. Low APE1 expression associated with aggressive phenotypes, poor survival and resistance to endocrine therapy in patients [30]. Taken together the data suggest a complex role for *APE1* in human tumours. We speculate that low APE1 in gliomas may promote a mutator phenotype where accelerated mutagenesis may promote aggressive cancers [40]. Similar to *APE1*, low *PTEN* mRNA was also associated with poor survival in our study and is consistent with previous observations in gliomas [31, 32]. At the protein levels, we found that nuclear *PTEN* was associated with adverse prognosis but no significant associations were evident for *PTEN* cytoplasmic expression. The data suggests that besides the role of *PTEN* as a negative regulator of the anti-apoptotic PI3K/Akt pathway, the recently described nuclear DNA repair function of *PTEN* [22-24] may influence prognosis in brain tumours. Interestingly, a preclinical study suggested that *APE1* may regulate *PTEN* expression through erg-1 transcription factor [17]. APE1 knockdown by siRNA in HeLa cells resulted in significant reduction in *PTEN* levels [17]. To investigate whether such functional interactions operate in human gliomas we performed *APE1*/*PTEN* combined mRNA and protein expression analysis in our cohorts. A consistent observation at the mRNA and the protein level was that tumours with low *APE1*/low *PTEN* had the worst survival compared to tumours with high *APE1*/high *PTEN* expression. These new observations not only provide prognostic information but also suggest that low *APE1* or low *PTEN* glioma cells could be targeted for personalized therapy by synthetic lethality. For example, we have recently demonstrated that *APE1* deficient cells are sensitive to *ATM* kinase inhibitors [33] and *PTEN* deficient cells are sensitive to BER inhibitors [34].

In addition to low *APE1* and low *PTEN*, we also observed that low *NBN* mRNA was associated with poor survival. Defects in the *NBN* gene result in a rare autosomal recessive disorder known as Nijmegen breakage syndrome (NBS) characterised by microcephaly and a predisposition to cancers including gliomas. Whether adverse prognostic significance of low *NBN* mRNA seen is due to increased genomic instability and the consequent aggressive phenotype is not known. Moreover, we did not observe any association at the protein level. However, it is important to note that, in a previous small study of 26 glioblastoma patients *NBN* was overexpressed in tumour tissue compared to adjacent normal tissue but was also not associated with survival [35]. In contrast, in head and neck cancer and in myelodysplastic syndrome high *NBN* appears to be associated with poor survival [36, 37]. However, a limitation of our immunohistochemistry study is that it is small cohort and retrospective. Larger prospective investigations are required to confirm these observations. Taken together, the data suggests that low expression of *APE1, NBN* and *PTEN* may increase genomic instability, leading to a mutator phenotype [14] that could promote accelerated accumulation of mutations leading to an aggressive glioma phenotype.

Previous studies have stratified high grade glioma patients in to prognostic groups based on global gene expression patterns. For example, Phillips et al identified three distinct molecular signatures in high grade glioma: Proneural, Proliferative and Mesenchymal. Patients with disease classified as ‘Proneural’ had a significantly better prognosis than patients with Proliferative or Mesenchymal disease. Proneural disease was more commonly seen in grade 3 gliomas, and on recurrence disease status was found to convert from Proneural to Mesenchymal status. Interestingly this study also found that a two gene model, incorporating PTEN and delta-like ligand 3 (DLL3), could predict survival. In this model patients with low PTEN expression had poor survival and those with high PTEN expression could be stratified according to DLL3 status [38]. Sturm et al describe six distinct methylation clusters in GBM patients. In this study isocitrate dehydrogenase 1 (IDH1) mutation was found to be associated with improved survival and with the Proneural classification. Patients with the G34 mutation of H3 histone, family 3A (H3F3A) had a better prognosis than patients that carried the K27 mutation [39]. Taken together the results from these, and other studies, suggest that there is significant heterogeneity within the high grade glioma population and that patients can be stratified in to differing prognostic groups based on this data.

This is the first study to report a DNA repair based prognostic index in glioblastomas. The DNA repair prognostic index incorporating *APE1* mRNA, *NBN* mRNA, *PTEN* mRNA, *PMS2* mRNA and *MGMT* mRNA expression separated patients into three distinct prognostic groups with worsening survival in the Test set and was further validated in the large TCGA dataset. The data not only suggest a DNA repair gene dose dependent biological effect in glioma patients but also implies that stratification could be employed for personalization of therapy. For example, we would suggest that patients in the worst prognostic group 3 could be spared aggressive toxic therapy that negatively impact quality of life outcomes. Alternatively such patients in the prognostic group 3 could be offered a personalized trial strategy. We acknowledge that the current study is retrospective but our data provides a platform for future prospective investigation. Moreover, whether such stratification could also be achieved at the protein level would require large multicentre studies with larger cohort of patients.

In addition to the prognostic significance of our five DNA repair genes of interest, our exploratory analysis stratifying patients by treatment group suggests that they may also have a potential role as predictive markers. Due to limitations of the data available we were only able to classify patients as to whether they have received chemotherapy or radiotherapy during the course of their treatment. Our data at the mRNA level demonstrates that *APE1, MGMT, PTEN* and *PMS2* were only associated with survival in patients that received radiotherapy during the course of their treatment. Similarly *APE1, MGMT*, and *PMS2* mRNA expression levels were only associated with survival in the chemotherapy treated group. While these results are interesting caution should be exerted in their interpretation given that the non-treatment subgroups are small. It is also difficult to draw conclusions from the subgroup analysis in the Nottingham Cohort as the numbers are small. For example, while 52 patients were known to have received radiotherapy in the Nottingham Cohort only two patients were recorded as not receiving radiotherapy.

An additional novel feature of our study is that we have conducted the first artificial neural network based interaction modelling of DNA repair genes in the TCGA cohort. The aim of this was to identify genes that interacted with the key DNA repair genes identified earlier and to identify the most influential genes (hubs) in the DNA repair system. The advantage of the ANN approach was that it modelled using non-linear functions and was not constrained by reliance on linear mathematics [40]. We focussed this interactomic study on *APE1* mRNA, *NBN* mRNA, *PTEN* mRNA, *PMS2* mRNA and *MGMT* mRNA interactions. Several key hubs with potential roles in glioma pathogenesis were identified. As additional validation we incorporated these hubs in a multivariate model which also included *APE1, NBN, PTEN, PMS2* and *MGMT*. We found that *FOXG1, THRA, RFX*4, *STXBP6, HPRT1, DCLK2* and *TOGLN2* were independently prognostic along with *APE1, NBN, PTEN, PMS2* and *MGMT*. Reassuringly, *FOXG1, THRA*, STXBP6 and *RFX*4 genes have previously been reported to be involved in glioma pathogenesis [41-43]. In fact *FOXG1* (Forkhead box protein G1), the most significantly associated independent variable in the current study, is a key transcriptional repressor protein with essential roles in brain development. Germ-line mutation in the *FOXG1* gene has been associated with atypical Rett syndrome characterised by microcephaly and psychomotor symptoms [41]. More importantly, a recent study has provided compelling pre-clinical evidence for the role of *FOXG1* in glioblastoma growth. *FOXG1* knockdown resulted in impaired glioblastoma growth *in vitro* and *in vivo* in that study [44]. Interestingly, the thyroid hormone receptor (*THRA*) axis has also been shown to be essential for glioblastoma growth [42]. *RFX*4 (regulatory factor X 4), a transcription factor known to influence HLA Class II expression is overexpressed in gliomas compared to normal brain tissues [43]. In another study, STXBP6 (syntaxin binding protein 6-amisyn) that is known to be involved in vesicle-mediated intracellular transport was found to be differentially expressed in high versus low grade gliomas [45]. Taken together the data not only validates the ANN approach utilized in the current study but also suggests a complex interaction between DNA repair, transcription and other essential cellular processes in glioma pathogenesis.

Given the essential role of *APE1, NBN, PTEN, PMS2* and *MGMT* in adult tumours we explored if these genes also influence outcomes in paediatric high grade gliomas/glioblastomas. Previous studies have demonstrated that, at the molecular level, paediatric high grade glioma differ from adult tumours [46]. For example, *PTEN* mutations are relatively common in adult high grade glioma but are less common in childhood tumours [47]. *MGMT* expression also varies between adult and paediatric gliomas; whereas more than 85% of paediatric patients demonstrate normal or low *MGMT* levels [48], in adult tumours only 50% show low *MGMT* expression [7]. These molecular differences imply that the pathogenesis of childhood tumours differs from that seen in adult glioma and may explain why the results from our paediatric cohort vary from that seen in our adult cohort. However, in common with their adult counterparts, earlier studies have shown that the small numbers of children with *PTEN* mutated tumours have a poor prognosis [48]. In addition, childhood tumours with a methylated *MGMT* promoter have previously been shown to have a higher average survival than those without promoter methylation [48]. As our paediatric cohort is small, and *PTEN* mutation and variation in *MGMT* expression is relatively uncommon, it is conceivable that we were not able to detect any association with survival due to sample size. Interestingly in our cohort there is a trend towards low *PTEN* expression being associated with poor survival, although this does not retain significance after correction for multiple comparisons. Larger scale studies are required to clarify the role of DNA repair in paediatric high grade glioma.

In conclusion, our study suggests that multiple DNA repair pathways may operate to influence biology and clinical outcomes in high grade adult gliomas. *APE1, NBN, PTEN, PMS2* and *MGMT* combined prognostication could allow stratification and personalization of therapy.

## MATERIALS AND METHODS

### Adult Glioblastoma gene expression data sets

#### Test set

The Test set (E-GEOD-13041) was obtained from http://www.ebi.ac.uk/arrayexpress/ and is a publically available gene expression dataset for patients with a diagnosis of GBM. This dataset contained microarray gene profiling data for 267 patients using 3 different Affymetrix platforms. 191 GBM patients were included in the subsequent data analysis for the Test dataset, all of whom were profiled using the Affymetrix U133A array. The median age of patients in the Test dataset was 54 years (range 18-86 years). 73/191 (38.2%) of patients were female. Patients were followed up for a median of 385 days (range 7-3353 days) and at the end of follow-up 176/191 (92.1%) had died.

#### Validation set

The Validation dataset was downloaded from ‘The Cancer Genome Atlas (TCGA)’ (http://cancergenome.nih.gov/) for patients with a diagnosis of GBM with gene expression data assessed using the HT_HG_U133A Affymetrix array. 548 files were identified in this dataset. Duplicate cases were removed alongside cases with missing survival data or identified as not meeting the original study criteria. A total of 508 cases were included in the subsequent analyses. In the TCGA dataset the median age of patients was 59 years (range 10-89 years). 200/508 (39.4%) of patients were female. Karnofsky performance status (KPS) data was available for 381 patients; median KPS was 80 (range 20-100). 416/508 (81.9%) of patients had died after a median follow up time of 353 days (range 2-3880 days). 351 (69.1%) were treated with chemotherapy; 294 (57.9%) received temozolomide. Baseline demographic data for the TCGA dataset is shown in Supplementary Table S15.

### Bioinformatics

#### DNA repair gene association studies

We investigated the clinical significance of 188 DNA repair genes (Supplementary Table S16) in the Test and TCGA datasets. A total of 157 DNA repair genes, represented by 248 probes, were present in both datasets and included in subsequent analyses. Baseline demographic data was also extracted including: age, gender, performance status, and treatment and survival data (if available). Xtile (version 3.6.1, Yale University, USA) was used to dichotomise (high/low) levels of DNA gene expression prior to Kaplan Meier survival analysis. Kaplan Meier survival curves were constructed in SPSS (Version 20, Chicago, USA) for 248 probes (in both the Test and TCGA datasets) and a log rank score calculated. The Benjamini and Hochberg False Discovery Rate calculation (BH FDR) [49] was applied to account for multiple comparisons. Cox multivariate regression models were constructed for each dataset including probes significant (with BH FDR correction) in both datasets (n=14). Non-significant probes after the first round of analysis were removed and the analysis re-run. This was repeated until only significant probes remained (6 probes in the Test dataset and 7 probes in the TCGA dataset). The models for the two datasets were compared and the analysis re-run with the five probes significant in both datasets.

### DNA repair prognostic index

Prognostic indices were calculated for each dataset (PI_1=prognostic index for the Test dataset and PI_2=prognostic index for the TCGA dataset) using the following equation:

Σ (DNA repair gene expression level* β value)

where DNA repair gene expression is represented as 0 (low) or 1 (high) and the β value is obtained from the final multivariate model described above. A combined prognostic index (PI_3) was also calculated using the mean β value for each gene from the two datasets. The prognostic index calculated from each dataset (PI_1 and PI_2) as well as the combined prognostic index (PI_3), was then tested in both the Test and TCGA datasets. Patients were divided into 3-4 prognostic groups based on their prognostic score and Kaplan Meier survival curves were constructed. The log rank test was applied to assess the survival difference between groups. The combined prognostic index (PI_3) separated patients into 3 statistically significant prognostic groups in the Test and TCGA datasets.

Using PI_3, curves were constructed to predict 1, 2 and 3 year survival in GBM patients in both the Test and TCGA datasets. Firstly, Kaplan Meier survival life tables were analysed to determine the percentage of patients alive at 1, 2 and 3 years. The percentage survival at 1 year (y axis) was plotted against the median prognostic score for patients within each of the 3 prognostic groups (x axis) and a 2^nd^ order polynomial curve fitted to the data. This process was repeated for 2 and 3 year survival.

### Artificial neural network (ANN) analysis

Artificial neural network (ANN) analysis was used to identify genes that interact with *PTEN, APE1, NBN, MGMT* and *PMS2* in the TCGA dataset. The probes selected to represent each gene were those used in the prognostic index (*PTEN* 204054_at, *APE1* 210027_s_at, *NBN* 202905_x_at, *MGMT* 204880_at and *PMS2* 209805_at). A total of 22,277 probes were screened to identify those best able to predict *PTEN, APE1, NBN, MGMT* or *PMS2* expression. The technique used was a non-linear, ANN modelling based, data mining approach which employed supervised learning with a multilayer perception architecture modified with a sigmoidal transfer function. The model weights were updated after each epoch by a feed forward back propagation algorithm. A Monte-Carlo cross validation strategy was employed prior to ANN training, where the samples were randomly segregated in to three subsets; 60% for training, 20% for testing and 20% for validation of model performance for 50 bootstraps [15]. The network momentum and learning rate were respectively set as 0.1 and 0.5. Two hidden nodes were utilised. The output node was coded as 0 if a case was low *PTEN, APE1, NBN, MGMT* or *PMS2* expression (<median) and 1 if high *PTEN, APE1, NBN, MGMT* or *PMS2* expression (>median). Inputs were ranked in ascending order based on their classification error. The top 200 predictive genes identified were then applied to an ANN based network inference algorithm as described in earlier studies [16]. This model predicted a weighted link (direction and magnitude) between each of the top 100 gene probe markers associated with *PTEN, APE1, NBN, MGMT* or *PMS2* expression. The 100 strongest pairwise interactions were then visualised as a map with Cytoscape (Version 3.0.1, Cytoscape Consortium, San Diego, USA) [50].

In a second bioinformatics analysis, we sought to obtain a robust ranking of genes that are differentially expressed between the mRNA *APE1*+, *NBN*+, *PTEN*+, *MGMT*+ or *PMS2* + cases and the mRNA *APE1*−, *NBN*−, *PTEN*−, *MGMT*− or *PMS2*− and have high predictive power, by applying an ensemble sample classification method within a leave-one-out cross-validation scheme. For this purpose, the 508 patient samples were first grouped into 508 different training/test set partitions, using 507 samples for the training sets and the remaining sample as the test set. For each of the 507 training sets differentially expressed genes were selected independently with the “Empirical Bayes moderated t-statistic” [51] and used to train a machine learning model, which was evaluated based on the left-out sample (a procedure known as “external cross-validation”). To classify the left-out sample, the prediction results of four algorithms (Support Vector Machine, Random Forest, kNN and Prediction Analysis for Microarrays, with all parameters being optimised by using a grid search within a nested cross-validation) [52] were combined to a majority-vote ensemble classifier as to compensate for the inevitable inherent biases and variances that exists amongst each of these machine learning algorithms. In order to rank the genes based on the cross-validation results, their frequency of occurrence in the list of significantly differentially expressed genes (p value < 0.05) across different cross-validation cycles was recorded, and genes received higher scores the more often they had been selected. All steps of the analysis were conducted using an in-house web-application for microarray analysis, available at www.arraymining.net.

Supplementary Figure 3 summarises the methods used to develop the prognostic index and for ANN analysis.

### Evaluation of *APE1, NBN, PTEN* and *PMS2* protein expression

Investigation of the protein expression of *APE1, NBN, PTEN* and *PMS2* in high grade glioma was carried out on paraffin-embedded tumour sections from 61 high grade glioma patients treated at Nottingham University Hospitals (NUH) between 2005 and 2011. Forty-three (70.5%) high grade patients were male and 19 patients (31.1%) were alive at the end of the study. Eighteen patients (29.5%) had been diagnosed with a glioma prior to inclusion in the study; 4 (6.6%) had already received radiotherapy and 3 (4.9%) had previously received chemotherapy. Median age at trial histology was 54 years (range 22-81 years). In total 52 (85.2%) patients were known to have received radiotherapy during the course of their illness and 39 (63.9%) were known to have received chemotherapy. The median number of lines of chemotherapy given was one (range 0-4). Baseline demographic information for the Nottingham Cohort is shown in Supplementary Table S17. The immunohistochemistry study has been approved by the Regional Ethics Committee (Reference number 08/H0406/102). An additional 18 patients with low grade (grade 2) glioma were also stained for *APE1, NBN, PTEN* and *PMS2*.

Immunohistochemical staining was performed using the Leica Novolink max polymer detection system and Thermo Scientific Shandon Sequenza chambers as per the manufacturer's instructions. Slides were heated to 60°C prior to passage through xylene to deparaffinise and then rehydration in decreasing concentrations of alcohol. Citrate buffer (pH 6) heated to 95°C (20 minutes) was used for antigen retrieval. All sections were incubated with primary antibody (*APE1* 1:300 [Novus Biologicals], *NBN* 1:150 [Sigma], *PTEN* 1:150 [Cell Signalling, clone D4.3] and *PMS2* 1:200 [BD Pharmingen, clone A16-4]) for 1 hour at room temperature. 3,3’ – Diaminobenzidine (DAB) was used as a chromogen. All sections were counterstained with haematoxylin. Slides were dehydrated in increasing concentrations of alcohol and passed through xylene prior to mounting. A negative control was performed by omission of the primary antibody and control sections were included in each run.

Tumour sections were evaluated by a Histopathologist (TA) blinded to the clinicopathological characteristics of the individual. A representative section of the slide was scored and the intensity of nuclear or cytoplasmic staining grouped as follows: 0 = no staining, 1 = weak staining, 2 = moderate staining and 3 = strong staining. The percentage of each staining category was estimated, the values summed and an H score calculated (range 0-300). Two patients had more than one pathology specimen from the same procedure and therefore an average H score was calculated.

### Statistical analysis of immunohistochemistry data

Baseline demographic data were collected, in addition to treatment and survival information. Data analysis was performed in SPSS (Version 20, Chicago, USA). Categorical variables are expressed as number and percentage and continuous variables as median and range. Xtile (Version 3.6.1, Yale University, USA) was used to dichotomise H-score expression of *APE1* (low ≤160, high >160), *NBN* (low ≤85, high >85), nuclear *PTEN* (low ≤50, high >50), cytoplasmic *PTEN* (low ≤10, high >10) and *PMS2* (low ≤120, high >120) into high and low expression. Kaplan Meier survival curves were constructed for each marker and the log rank test used to determine the survival difference between groups. Chi squared tests (with or without Yates’ continuity correction, as appropriate) were used to assess the association between two categorical variables. Mann Whitney U test was used to compare continuous, non-normally distributed variables between two groups. Cox multivariate analysis was performed to determine independent predictors of survival. Statistical significance was defined as p≤0.05.

### Cells lines, tissue culture and Western blot analysis

LN229 and LN18 human glioma cell lines were purchased from ATCC and were grown in Dulbecco's Modified Eagle Medium (DMEM with 4500mg/L glucose, L-glutamine, sodium pyruvate) with the addition of 5% foetal bovine serum. 5ml of 1% penicillin/streptomycin (10,000 units penicillin and 10mg streptomycin/mL) was added to the media. All media and additives were purchased from Sigma, UK. To evaluate the specificity of the antibodies used for the immunohistochemical study cell lysates were prepared and Western blot analysis performed. Primary antibodies were incubated at room temperature for 1 hour (*APE1* 1:1000 dilution, *NBN* 1:500 dilution, *PTEN* 1:500 dilution, *PMS2* 1:500 dilution and *beta actin* 1:10000 dilution [Abcam] or *beta tubulin* 1:2000 [Abcam]). Infrared dye-labelled secondary antibodies (Li-Cor) [IRDye 800CW Donkey Anti-Rabbit IgG and IRDye 680CW Donkey Anti-Mouse IgG] were incubated at a dilution of 1:10000 for 1 hour. Membranes were scanned with a Li-Cor Odyssey machine (700 and 800nm) to determine protein expression.

### Paediatric high-grade glioma and glioblastoma gene expression data analysis

Two independent microarray gene expression data sets of paediatric high-grade glioma (pHGG) and paediatric glioblastoma (pGBM) were used in this analysis. The pHGG dataset (GSE19578) contained mRNA expression profiles from 53 pHGG patients with median age of 9.9 years (range from 0 to 23 years) and the median follow-up of 1.2 years (range from 0.1 to 7.6 years). The pGBM dataset (GSE34824) contained expression profiles from tumour samples of 27 pGBM patients with the median age of 11.5 years (range from 2 to 20 years). 16/27 (59.3%) of pGBM patients were male. The median overall survival of these 27 pGBM patients was 12 months (range from 5 to 55 months). In both datasets, Affymetrix HG-U133 Plus arrays were used for the mRNA profiling. The raw data was downloaded from the GEO database and pre-processed with robust multi-array average (RMA) algorithm. Probesets that are ‘absent’ (present / absent call using MAS5) in all samples were removed from the analysis and the remaining probesets were mapped to Entrez GeneID using Bioconductor annotation package. Normalized expression values for 16 probesets (mapped to 5 DNA repair genes) were extracted and tested for survival using the univariate Cox proportional hazards regression model. The most significant probeset from the regression model for *APE1, MGMT, NBN, PMS2* and *PTEN* was selected and the Kaplan Meier survival analysis was performed with expression levels dichotomised in to low/high expression at the median.

## SUPPLEMENTARY MATERIAL TABLES AND FIGURES


