# Supplementary material for: Dissecting DNA repair in adult high grade gliomas for patient stratification in the post-genomic era
Source: Oncotarget. 2014 Jul 9;5(14):5764–81. doi: 10.18632/oncotarget.2180 (PMC4170616; doi:10.18632/oncotarget.2180)
Supplement: Supplementary file 1 [file oncotarget-05-5764-s001.pdf]

Dissecting DNA repair in adult high grade gliomas for patient stratification in the post-genomic era

Supplementary Material

Supplementary Table S1: Equations representing predictive curves for 1, 2 and 3 year survival based on PI3 in both the Test and TCGA datasets.

Survival	Equation (Test dataset)	Equation (TCGA dataset)
1 year	$Y=48.95 + (-44.19*X) + (-17.48*X^2)$	$Y=49.69 + (-34.77*X) + (0.8499*X^2)$
2 years	$Y=11.25 + (-30.22*X) + (-24.67*X^2)$	$Y=12.77 + (-23.69*X) + (24.89*X^2)$
3 years	$Y=6.272 + (-20.85*X) + (-22.76*X^2)$	$Y=5.020 + (-11.20*X) + (28.91*X^2)$

Supplementary Table S2: The association between *APE1*, *PMS2*, *NBN*, *MGMT* and *PTEN* expression and age in the TCGA and Nottingham Cohorts

**Gene**		**TCGA dataset (mRNA)**		**Nottingham Cohort (Protein)**	
		**Median age (years)**	***P* value**	**Median age (years)**	***P* value**
APE1	Low	60	**<0.001**	45.5	0.979
	High	53		49.5	
PMS2	Low	59	**0.016**	45.5	0.709
	High	61		47.5	
NBN	Low	60	0.218	45	0.914
	High	59		48.5	
MGMT	Low	60.5	0.372	N/A	N/A
	High	59		N/A	
PTEN	Low	61	**<0.001**	49	0.222
	High	53		43	

Significant p values (≤0.05) are shown in bold.

Supplementary Table S3: Baseline demographic data for pediatric high-grade glioma (pHGG) and pediatric glioblastoma (pGBM) datasets

**pHGG cohort (GSE19578)**	
**Variable**	**Number**
Diagnosis	
Anaplastic Astrocytoma	9
Anaplastic Oligodendroglioma	5
Anaplastic PXA[1]	2
Glioblastoma	33
Glioblastoma variant	4
Grade	
III	16
IV	37
Surgical resection	
> 90% debulking	16
< 90% debulking	30
Adjuvant therapy	
Chemotherapy (CT)	8
Radiotherapy (RT)	5
CT + RT	31
Survival status	
Alive	7
Deceased	40
**pGBM cohort (GSE19578)**	
Gender	16
Male	10
Female	
Survival status	
Alive	4
Deceased	16
Recurrence	
No	4
Yes	12

[1] PXA = pleomorphic xanthoastrocytoma

Supplementary Table S4: Univariate Cox proportional hazard regression of survival for pediatric high-grade glioma (pHGG) and pediatric glioblastoma (pGBM) patients

		**pHGG (n = 53)**		**pGBM (n = 27)**	
**Gene**	**Probeset ID**	**HR (CI)**	**P**	**HR (CI)**	**P**
APEX1	210027_s_at	0.98 (0.46, 2.09)	0.9587	0.94 (0.51, 1.72)	0.844
MGMT	204880_at	1.13 (0.75, 1.72)	0.5546	0.94 (0.51, 1.72)	0.844
NBN	202906_s_at	2.52 (1.11, 5.73)	0.0278	1.03 (0.31, 3.38)	0.9597
	217299_s_at	1.95 (1.02, 3.73)	0.0439	0.66 (0.20, 2.22)	0.5005
	240510_at	1.99 (0.94, 4.19)	0.0717	0.20 (0.01, 3.89)	0.2886
	202905_x_at	1.45 (0.72, 2.90)	0.2988	0.92 (0.15, 5.64)	0.927
	202907_s_at	0.75 (0.39, 1.44)	0.3894	1.41 (0.38, 5.23)	0.6065
PMS2	209805_at	2.01 (0.94, 4.29)	0.0721	2.59 (0.50, 13.31)	0.2539
PTEN	225363_at	0.56 (0.30, 1.04)	0.0662	0.91 (0.55, 1.51)	0.7241
	242622_x_at	1.62 (0.77, 3.41)	0.2073	0.57 (0.24, 1.37)	0.2094
	228006_at	0.72 (0.43, 1.23)	0.2331	0.94 (0.61, 1.44)	0.7646
	204053_x_at	1.34 (0.81, 2.23)	0.2523	0.98 (0.55, 1.73)	0.9347
	227469_at	0.42 (0.07, 2.66)	0.3592	2.66 (0.06, 120.21)	0.6145
	233254_x_at	1.18 (0.53, 2.63)	0.694	0.66 (0.19, 2.29)	0.5106
	211711_s_at	1.11 (0.66, 1.87)	0.7046	0.89 (0.54, 1.46)	0.6359
	204054_at	1.00 (0.53, 1.87)	0.9941	1.06 (0.59, 1.93)	0.839

HR: hazard ratio, *CI*: 95% confidence interval

P: Wald test p-value from fitting a univariate Cox regression model

Supplementary Table S5: Gene names and functions represented in the APE1 interactome

Gene ID	Gene name	Biological processes
ACACB	ACETYL-COA CARBOXYLASE 2	Coenzyme metabolic process; gluconeogenesis; fatty acid biosynthetic process
AMPD3	AMP DEAMINASE 3	Purine nucleobase metabolic process
AP4S1	AP-4 COMPLEX SUBUNIT SIGMA-1	Intracellular protein transport; receptor-mediated endocytosis
C14orf1	ERGOSTEROL BIOSYNTHETIC PROTEIN 28-RELATED	Unknown
C2CD2	C2 CALCIUM-DEPENDENT DOMAIN CONTAINING 2	Unknown
CCDC69	COILED-COIL DOMAIN-CONTAINING PROTEIN 69	Unknown
CCNB1IP1	E3 UBIQUITIN-PROTEIN LIGASE CCNB1IP1	Metal ion binding; ligase activity
CCT3	T-COMPLEX PROTEIN 1 SUBUNIT GAMMA	Protein folding; protein complex assembly; protein complex biogenesis
CDC34	UBIQUITIN-CONJUGATING ENZYME E2 R1	Apoptotic process; mitosis; meiosis; chromosome segregation; dorsal/ventral axis specification; dorsal/ventral axis specification; embryo development; apoptotic process; negative regulation of apoptotic process
CDKN2C	CYCLIN-DEPENDENT KINASE 4 INHIBITOR C	Metabolic process; cell cycle; regulation of catalytic activity
CFLAR	CASP8 AND FADD-LIKE APOPTOSIS REGULATOR	Apoptotic process; proteolysis; apoptotic process; regulation of catalytic activity
CLPP	ATP-DEPENDENT CLP PROTEASE PROTEOLYTIC SUBUNIT, MITOCHONDRIAL-RELATED	Proteolysis
CREB1	CYCLIC AMP-RESPONSIVE ELEMENT-BINDING PROTEIN 1	Immune system process; transcription from RNA polymerase II promoter; cell communication; neurological system process; ectoderm development; nervous system development; response to stress; regulation of transcription from RNA polymerase II promoter
CREBL2	CAMP-RESPONSIVE ELEMENT-BINDING PROTEIN-LIKE 2	Sequence-specific DNA binding; sequence-specific DNA binding transcription factor activity
CYLD	UBIQUITIN CARBOXYL-TERMINAL HYDROLASE CYLD	Translation
DDX49	ATP-DEPENDENT RNA HELICASE DDX49-RELATED	Nucleobase-containing compound metabolic process; translation; regulation of translation

DHX9	ATP-DEPENDENT RNA HELICASE A	mRNA splicing
ECSIT	EVOLUTIONARILY CONSERVED SIGNALING INTERMEDIATE IN TOLL PATHWAY, MITOCHONDRIAL	Oxidoreductase activity, acting on NAD(P)H and sequence-specific DNA binding transcription factor activity
EEF1G	ELONGATION FACTOR 1-GAMMA	Immune system process; translation; cell communication; response to toxic substance; regulation of translation
EIF2S1	EUKARYOTIC TRANSLATION INITIATION FACTOR 2 SUBUNIT 1	Translation; regulation of translation
EPHB3	EPHRIN TYPE-B RECEPTOR 3	Nervous system development
ETV1	ETS TRANSLOCATION VARIANT 1	B cell mediated immunity; macrophage activation; transcription from RNA polymerase II promoter; cell cycle; cell communication; mesoderm development; haemopoiesis; cellular defence response; regulation of transcription from RNA polymerase II promoter
EXOSC4	EXOSOME COMPLEX COMPONENT RRP41 (PTHR11953:SF0)	tRNA metabolic process; RNA catabolic process; rRNA metabolic process
FICD	ADENOSINE MONOPHOSPHATE-PROTEIN TRANSFERASE FICD	ATP binding ; protein adenylyltransferase activity
FOXG1	FORKHEAD BOX PROTEIN G1	Carbohydrate metabolic process; transcription from RNA polymerase II promoter; cell cycle; visual perception; sensory perception; segment specification; anterior/posterior axis specification; segment specification; anterior/posterior axis specification; ectoderm development; mesoderm development; anatomical structure morphogenesis; embryo development; cell differentiation; nervous system development; regulation of transcription from RNA polymerase II promoter; cellular component organization
FYCO1	FYVE AND COILED-COIL DOMAIN-CONTAINING PROTEIN 1	Protein binding; metal ion binding
GNS	N-ACETYLGLUCOSAMINE-6-SULFATASE	Sulphur compound metabolic process; phospholipid metabolic process; polysaccharide metabolic process; phospholipid metabolic process
GOLGB1	GOLGIN SUBFAMILY B MEMBER 1	Protein binding
GPHN	GEPHYRIN	Protein metabolic process
HN1	HAEMATOLOGICAL AND NEUROLOGICAL EXPRESSED 1	Apoptosis
ITSN2	INTERSECTIN-2	Metabolic process; synaptic transmission; neurotransmitter secretion; intracellular protein transport; endocytosis; regulation of catalytic activity
LSM7	U6 SNRNA-ASSOCIATED SM-LIKE PROTEIN LSM7	RNA splicing, via trans-esterification reactions; mRNA splicing, via spliceosome

MAPK1IP1L	MITOGEN-ACTIVATED PROTEIN KINASE 1 INTERACTING PROTEIN 1-LIKE	Unknown
METTL17	METHYLTRANSFERASE-LIKE PROTEIN 17, MITOCHONDRIAL	Protein complex assembly; cation transport; protein complex biogenesis
METTL3	N6-ADENOSINE-METHYLTRANSFERASE 70 KDA SUBUNIT	mRNA processing
MNAT1	CDK-ACTIVATING KINASE ASSEMBLY FACTOR MAT1	DNA repair; protein complex assembly; cell cycle; regulation of catalytic activity; protein complex biogenesis
MTHFD1	C-1-TETRAHYDROFOLATE SYNTHASE, CYTOPLASMIC	Metabolic process; cellular process
NFE2L1	NUCLEAR FACTOR ERYTHROID 2-RELATED FACTOR 1	Proteolysis; mesoderm development; haemopoiesis; response to stress
NREP	NEURONAL REGENERATION-RELATED PROTEIN	Neural function; cellular differentiation ; increases retinoic-acid regulation of lipid-droplet biogenesis; down-regulates the expression of TGFB1 and TGFB2 but not of TGFB3; potential role in the regulation of alveolar generation
OSGEP	TRNA THREONYLCARBAMOYLADENOSINE BIOSYNTHESIS PROTEIN OSGEP-RELATED	Proteolysis
PLEKHJ1	PLECKSTRIN HOMOLOGY DOMAIN-CONTAINING FAMILY J MEMBER 1	Phospholipid binding
POLR2F	DNA-DIRECTED RNA POLYMERASES I, II, AND III SUBUNIT RPABC2	DNA-directed RNA polymerase activity; protein kinase activity
PUF60	POLY(U)-BINDING-SPLICING FACTOR PUF60	DNA replication; RNA splicing, via trans-esterification reactions; transcription from RNA polymerase II promoter; mRNA splicing, via spliceosome; mRNA polyadenylation; RNA splicing, via trans-esterification reactions; protein metabolic process; cell cycle
RDH11	RETINOL DEHYDROGENASE 11	Cellular amino acid biosynthetic process; steroid metabolic process; visual perception; sensory perception
RPS10	40S RIBOSOMAL PROTEIN S10	Translation
RPS29	40S RIBOSOMAL PROTEIN S29	Structural constituent of ribosome; zinc ion binding
SNRPC	U1 SMALL NUCLEAR RIBONUCLEOPROTEIN C	U1 snRNA binding; protein homodimerization activity

TGOLN2	TRANS-GOLGI NETWORK INTEGRAL MEMBRANE PROTEIN 2	Intracellular protein transport; exocytosis
TK2	THYMIDINE KINASE 2, MITOCHONDRIAL	Purine nucleobase metabolic process ;pyrimidine nucleobase metabolic process
TMX1	THIOREDOXIN-RELATED TRANSMEMBRANE PROTEIN 1	Protein folding; cellular protein modification process
TNIP1	TNFAIP3-INTERACTING PROTEIN 1	Ubiquitin-specific protease activity; mitogen-activated protein kinase binding
TPCN1	TWO PORE CALCIUM CHANNEL PROTEIN 1	Cellular process; cation transport
UBA52	PROTEIN UBBP4-RELATED	Proteolysis
UBR7	E3 UBIQUITIN-PROTEIN LIGASE UBR7-RELATED	Ubiquitin-protein ligase activity
WFS1	WOLFRAMIN	ATPase binding; transporter activity

Supplementary Table S6: Gene names and functions represented in the PMS2 interactome

**Gene ID**	**Gene name**	**Biological processes**
ACSL4	LONG-CHAIN-FATTY-ACID--COA LIGASE 4	Immune system process; fatty acid metabolic process; lipid transport
ADAP1	ARF-GAP WITH DUAL PH DOMAIN-CONTAINING PROTEIN 1	Metabolic process; cell communication; cell adhesion; regulation of catalytic activity
AGK	ACYLGLYCEROL KINASE, MITOCHONDRIAL	Metabolic process; cellular process
AK4	ADENYLATE KINASE ISOENZYME 4, MITOCHONDRIAL	Purine nucleobase metabolic process; pyrimidine nucleobase metabolic process
ANXA7	ANNEXIN A7	Fatty acid metabolic process
ATF1	CYCLIC AMP-DEPENDENT TRANSCRIPTION FACTOR ATF-1	Immune system process; transcription from RNA polymerase II promoter; cell communication; neurological system process; ectoderm development; nervous system development; response to stress; regulation of transcription from RNA polymerase II promoter
BLM	BLOOM SYNDROME PROTEIN	DNA replication; DNA repair; DNA recombination; cell cycle
C7orf26	PROTEIN Y56A3A.31	Actin binding; myosin binding
CALD1	CALDESMON	Mitosis; cellular component morphogenesis; cellular component organization
CCDC93	COILED-COIL DOMAIN-CONTAINING PROTEIN 93	Unknown
CCL4	C-C MOTIF CHEMOKINE 4-RELATED	Immune response; cellular process; response to stimulus
CD55	COMPLEMENT DECAY-ACCELERATING FACTOR	Complement activation; proteolysis; cell communication; cell-cell adhesion; blood coagulation; lipid transport
CD69	EARLY ACTIVATION ANTIGEN CD69	B cell mediated immunity; natural killer cell activation; response to stimulus
CSF2RB	CYTOKINE RECEPTOR COMMON SUBUNIT BETA	Natural killer cell activation; cellular process; haemopoiesis; response to stimulus
CTSS	CATHEPSIN S	Antigen processing and presentation of peptide or polysaccharide antigen via MHC class II; proteolysis
CXCL12	STROMAL CELL-DERIVED FACTOR 1	Chemokine activity; receptor binding
DDX56	ATP-DEPENDENT RNA HELICASE DDX56-RELATED	Nucleobase-containing compound metabolic process; translation; regulation of translation
DEPTOR	DEP DOMAIN-CONTAINING MTOR-INTERACTING PROTEIN	Metabolic process; regulation of catalytic activity
EDIL3	EGF-LIKE REPEAT AND DISCOIDIN I-LIKE DOMAIN-CONTAINING PROTEIN 3	Immune system process; proteolysis; synaptic transmission; cell-cell adhesion; visual perception; sensory perception; ectoderm development; mesoderm

		development; skeletal system development; angiogenesis; nervous system development; heart development; blood coagulation; lipid transport; intracellular protein transport; endocytosis; vitamin transport; regulation of catalytic activity
EFR3A	PROTEIN EFR3 HOMOLOG A	Role in hearing
EIF2AK1	EUKARYOTIC TRANSLATION INITIATION FACTOR 2-ALPHA KINASE 1	Immune system process; translation; protein phosphorylation; mitosis; response to stress
ENPP2	ECTONUCLEOTIDE PYROPHOSPHATASE/PHOSPHODIESTERASE FAMILY MEMBER 2	Nucleobase-containing compound metabolic process
ETV1	ETS TRANSLOCATION VARIANT 1	B cell mediated immunity; macrophage activation; transcription from RNA polymerase II promoter; cell cycle; cell communication; mesoderm development; haemopoiesis; cellular defence response; regulation of transcription from RNA polymerase II promoter
FAF2	FAS-ASSOCIATED FACTOR 2	Induction of apoptosis; cell communication; induction of apoptosis
FBXL18	F-BOX/LRR-REPEAT PROTEIN 18	Ubiquitination
GJB1	GAP JUNCTION BETA-1 PROTEIN	Cellular process; transport
GREM1	GREMLIN-1	Protein tyrosine kinase activator activity; cytokine activity
KLK6	KALLIKREIN-6	Proteolysis; response to stimulus
MAN1A1	MANNOSYL-OLIGOSACCHARIDE 1,2-ALPHA-MANNOSIDASE IA	Protein folding; proteolysis
MICALL2	MICAL-LIKE PROTEIN 2	Cellular component movement; cellular component morphogenesis; cellular component organization
MIOS	WD REPEAT-CONTAINING PROTEIN MIO	Role in oogenesis
MYLIP	E3 UBIQUITIN-PROTEIN LIGASE MYLIP	Cytoskeletal protein binding; ubiquitin-protein ligase activity
NBL1	NEUROBLASTOMA SUPPRESSOR OF TUMORIGENICITY 1	Morphogen activity; BMP binding
NEK7	SERINE/THREONINE-PROTEIN KINASE NEK7	Phosphate-containing compound metabolic process; protein phosphorylation
NINJ2	NINJURIN-2	Potential role in nerve regeneration and formation and function of other tissues
OBSL1	OBSCURIN-LIKE PROTEIN 1	Protein phosphorylation; cell communication; cell adhesion; muscle contraction; mesoderm development; muscle organ development; regulation of catalytic activity
OSTF1	OSTEOCLAST-STIMULATING FACTOR 1	SH3 domain binding
PMS2	MISMATCH REPAIR ENDONUCLEASE PMS2	Phosphate-containing compound metabolic process; nitrogen compound metabolic process; catabolic process; DNA repair; cellular process; response to stress

POLR2K	DNA-DIRECTED RNA POLYMERASES I, II, AND III SUBUNIT RPABC4	Respiratory electron transport chain; transcription from RNA polymerase II promoter
POU3F2	POU DOMAIN, CLASS 3, TRANSCRIPTION FACTOR 2	Transcription from RNA polymerase II promoter; regulation of transcription from RNA polymerase II promoter
PPP3CB	SERINE/THREONINE-PROTEIN PHOSPHATASE 2B CATALYTIC SUBUNIT BETA ISOFORM	Glycogen metabolic process; transcription from RNA polymerase II promoter; mRNA processing; protein phosphorylation; cell cycle; cell communication; response to stress; regulation of carbohydrate metabolic process; regulation of nucleobase-containing compound metabolic process
PTER	PHOSPHOTRIESTERASE-RELATED PROTEIN	Metabolic process
PYCARD	APOPTOSIS-ASSOCIATED SPECK-LIKE PROTEIN CONTAINING A CARD	Apoptotic process; proteolysis; apoptotic process; regulation of catalytic activity
RAC1	RAS-RELATED C3 BOTULINUM TOXIN SUBSTRATE 1	Metabolic process; cell communication; intracellular protein transport; receptor-mediated endocytosis
RBM28	RNA-BINDING PROTEIN 28	DNA replication; RNA splicing, via trans-esterification reactions; mRNA splicing, via spliceosome; mRNA polyadenylation; RNA splicing, via trans-esterification reactions; rRNA metabolic process; protein metabolic process; cell cycle; neurological system process; ectoderm development
RBX1	E3 UBIQUITIN-PROTEIN LIGASE RBX1	Metabolic process; cytokinesis; mitosis
RNF216	E3 UBIQUITIN-PROTEIN LIGASE RNF216	Proteolysis
RPS6KA5	RIBOSOMAL PROTEIN S6 KINASE ALPHA-5	Protein phosphorylation; cell cycle; cell communication; ectoderm development; mesoderm development; skeletal system development; nervous system development
RTN2	RETICULON-2	Ectoderm development; nervous system development; intracellular protein transport
SERPINB1	LEUKOCYTE ELASTASE INHIBITOR	Proteolysis; regulation of biological process; regulation of catalytic activity
SGMS1	PHOSPHATIDYLCHOLINE:CERAMIDE CHOLINEPHOSPHOTRANSFERASE 1	Lipid metabolic process
SLC4A3	ANION EXCHANGE PROTEIN 3	Cellular process; cellular component morphogenesis; ion transport; cellular component organization
SPINT2	KUNITZ-TYPE PROTEASE INHIBITOR 2	Immune system process; proteolysis; blood coagulation; regulation of catalytic activity
SPOCK3	TESTICAN-3	Metabolic process; regulation of catalytic activity
STXBP6	SYNTAXIN-BINDING PROTEIN 6	Neurotransmitter secretion; intracellular protein transport; synaptic vesicle exocytosis
TEAD1	TRANSCRIPTIONAL ENHANCER FACTOR TEF-1	Transcription from RNA polymerase II promoter; regulation of transcription from RNA polymerase II promoter
TNFSF10	TUMOR NECROSIS FACTOR LIGAND SUPERFAMILY MEMBER 10	Immune response; induction of apoptosis; cell-cell signalling; induction of apoptosis; skeletal system development; haemopoiesis; cellular defence response

TNPO1	TRANSPORTIN-1	Protein targeting; nuclear transport
TPD52L1	TUMOR PROTEIN D53	Protein homodimerization activity; identical protein binding
VEGFA	VASCULAR ENDOTHELIAL GROWTH FACTOR A	Cell cycle; cell communication; angiogenesis; response to stress
WIPI2	WD REPEAT DOMAIN PHOSPHOINOSITIDE-INTERACTING PROTEIN 2	Catabolic process; cell communication; response to stress; response to external stimulus; organelle organization; cellular component biogenesis
ZNF12	ZINC FINGER PROTEIN 12	DNA binding; zinc ion binding

Supplementary Table S7: Gene names and functions represented in the NBN interactome

Gene ID	Gene name	Biological processes
ACSL4	LONG-CHAIN-FATTY-ACID--COA LIGASE 4	Immune system process; fatty acid metabolic process; lipid transport
ALDH5A1	SUCCINATE-SEMIALDEHYDE DEHYDROGENASE, MITOCHONDRIAL	Nucleobase-containing compound metabolic process; cellular amino acid metabolic process
ARHGEF12	RHO GUANINE NUCLEOTIDE EXCHANGE FACTOR 12	Phospholipid binding; Rho guanyl-nucleotide exchange factor activity
ARPC4	ACTIN-RELATED PROTEIN 2/3 COMPLEX SUBUNIT 4	Cellular component movement; cellular component organization
B3GNT2	UDP-GLCNAC:BETAGAL BETA-1,3-N-ACETYLGLUCOSAMINYLTRANSFERASE 2	Female gamete generation; lipid metabolic process; translation; protein glycosylation; cell communication; dorsal/ventral axis specification; dorsal/ventral axis specification
BANP	PROTEIN BANP	DNA binding; p53 binding
CAPZA2	F-ACTIN-CAPPING PROTEIN SUBUNIT ALPHA-2	Cellular process; cellular component morphogenesis; cellular component organization
CD55	COMPLEMENT DECAY-ACCELERATING FACTOR	Complement activation; proteolysis; cell communication; cell-cell adhesion; blood coagulation; lipid transport
CUL5	CULLIN-5	Induction of apoptosis; proteolysis; cell cycle; induction of apoptosis
DCAF6	DDB1- AND CUL4-ASSOCIATED FACTOR 6	Apoptotic process; nucleobase-containing compound metabolic process; cell communication; apoptotic process; regulation of nucleobase-containing compound metabolic process
DEDD	DEATH EFFECTOR DOMAIN-CONTAINING PROTEIN	Protein binding; DNA binding
DUSP3	DUAL SPECIFICITY PROTEIN PHOSPHATASE 3	Phosphate-containing compound metabolic process; cellular protein modification process; cellular process
EVI5	ECOTROPIC VIRAL INTEGRATION SITE 5 PROTEIN HOMOLOG	Metabolic process; cellular process; cellular component morphogenesis; intracellular protein transport; exocytosis; regulation of catalytic activity; cellular component organization
FKBP1A	PEPTIDYL-PROLYL CIS-TRANS ISOMERASE-RELATED	Cellular protein modification process; cellular process
FRMD4B	FERM DOMAIN-CONTAINING PROTEIN 4B	Cellular process; cellular component morphogenesis; cellular component organization
GOLGA2	GOLGIN SUBFAMILY A MEMBER 2	Protein binding
GOLIM4	GOLGI INTEGRAL MEMBRANE PROTEIN 4	Endosome to Golgi protein trafficking

GOLT1B	VESICLE TRANSPORT PROTEIN GOT1B	Intracellular protein transport
GRIA3	GLUTAMATE RECEPTOR 3	Neuron-neuron synaptic transmission; neurological system process; cation transport
GSN	GELSOLIN	Cellular process; cellular component morphogenesis; cellular component organization
GTF2A1	TRANSCRIPTION INITIATION FACTOR IIA SUBUNIT 1	Transcription from RNA polymerase II promoter
HIPK3	HOMEODOMAIN-INTERACTING PROTEIN KINASE 3	Protein phosphorylation
HNRNPUL 2	HCG2044799-RELATED	Nuclei acid binding
ID4	DNA-BINDING PROTEIN INHIBITOR ID-4	Transcription from RNA polymerase II promoter; regulation of transcription from RNA polymerase II promoter
JUP	JUNCTION PLAKOGLOBIN	Female gamete generation; nitrogen compound metabolic process; biosynthetic process; transcription from RNA polymerase II promoter; cell-cell signalling; cell adhesion; pattern specification process ;pattern specification process; cellular component morphogenesis ;embryo development; cell differentiation; heart development; response to stimulus; protein localization; regulation of transcription from RNA polymerase II promoter; cytoskeleton organization
KLF11	KRUEPPEL-LIKE FACTOR 11	B cell mediated immunity; transcription from RNA polymerase II promoter; anterior/posterior axis specification; anterior/posterior axis specification; mesoderm development; system development; response to stimulus; regulation of transcription from RNA polymerase II promoter
KPNA4	IMPORTIN SUBUNIT ALPHA-4	Protein targeting; nuclear transport
LRRFIP1	LEUCINE-RICH REPEAT FLIGHTLESS-INTERACTING PROTEIN 1	Transcription from RNA polymerase II promoter
LYST	LYSOSOMAL-TRAFFICKING REGULATOR	Cell communication; protein targeting
MAGI2	MEMBRANE-ASSOCIATED GUANYLATE KINASE, WW AND PDZ DOMAIN-CONTAINING PROTEIN 2	Metabolic process; synaptic transmission; neurological system process
MBD4	METHYL-CPG-BINDING DOMAIN PROTEIN 4 (	DNA repair
MED1	MEDIATOR OF RNA POLYMERASE II TRANSCRIPTION SUBUNIT 1	Transcriptional co-activator activity; chromatin binding
MMP16	MATRIX METALLOPROTEINASE-16	Proteolysis
MYO10	UNCONVENTIONAL MYOSIN-X	Metabolic process; cytokinesis; cellular component movement; mitosis; cell communication; cellular component morphogenesis; muscle organ development; intracellular protein transport; vesicle-mediated transport; regulation of catalytic activity; cellular component organization
NBN	NIBRIN	DNA repair; DNA recombination

NCAM1	NEURAL CELL ADHESION MOLECULE 1	Immune system process; induction of apoptosis; cellular protein modification process; cell cycle; cell-cell signalling; cell-cell adhesion; muscle contraction; neurological system process; ectoderm development; mesoderm development; induction of apoptosis; angiogenesis; nervous system development; muscle organ development
NFAT5	NUCLEAR FACTOR OF ACTIVATED T-CELLS 5	Transcription from RNA polymerase II promoter; regulation of transcription from RNA polymerase II promoter
POU3F2	POU DOMAIN, CLASS 3, TRANSCRIPTION FACTOR 2	Transcription from RNA polymerase II promoter; regulation of transcription from RNA polymerase II promoter
PPFIA1	LIPRIN-ALPHA-1	Cellular process; cell adhesion; ectoderm development; nervous system development
PPFIBP1	LIPRIN-BETA-1	Potential regulator of disassembly of focal adhesions
PPP1R2	PROTEIN PHOSPHATASE INHIBITOR 2	Glycogen metabolic process; regulation of catalytic activity
PPP6C	SERINE/THREONINE-PROTEIN PHOSPHATASE 6 CATALYTIC SUBUNIT	Glycogen metabolic process; transcription from RNA polymerase II promoter; mRNA processing; protein phosphorylation; mitosis; cell communication; response to stress; regulation of carbohydrate metabolic process; regulation of nucleobase-containing compound metabolic process
PRRC2C	PROTEIN PRRC2C	Nucleobase-containing compound metabolic process
PTP4A2	PROTEIN TYROSINE PHOSPHATASE TYPE IVA 2	Protein phosphorylation; mitosis
PTPRA	RECEPTOR-TYPE TYROSINE-PROTEIN PHOSPHATASE ALPHA	Cellular protein modification process; cell communication
RAP2C	RAS-RELATED PROTEIN RAP-2C	Metabolic process; synaptic transmission; cell adhesion; neurological system process; intracellular protein transport; receptor-mediated endocytosis
RFX4	TRANSCRIPTION FACTOR RFX4	Transcription from RNA polymerase II promoter; tRNA metabolic process; regulation of transcription from RNA polymerase II promoter
RHBDD3	RHOMBOID DOMAIN-CONTAINING PROTEIN 3	Metabolic process
SCAF11	PROTEIN SCAF11	mRNA splicing, via spliceosome
SEMA6A	SEMAPHORIN-6A	Immune system process; cell communication; neurological system process; ectoderm development; mesoderm development; angiogenesis; nervous system development; heart development
SERPINB1	LEUKOCYTE ELASTASE INHIBITOR	Proteolysis; regulation of biological process; regulation of catalytic activity
SF1	SPLICING FACTOR 1	RNA splicing, via transesterification reactions; mRNA splicing, via spliceosome; RNA splicing, via transesterification reactions
SF3B1	SPLICING FACTOR 3B SUBUNIT 1	RNA splicing, via transesterification reactions; mRNA splicing, via spliceosome; RNA splicing, via transesterification reactions
SGPP1	SPHINGOSINE-1-PHOSPHATE PHOSPHATASE 1	Apoptotic process; phospholipid metabolic process; phospholipid metabolic process; cell communication; apoptotic process
SORT1	SORTILIN	Lipid metabolic process; lipid transport; intracellular protein transport; receptor-mediated endocytosis

SS18	PROTEIN SSXT	Nucleobase-containing compound metabolic process; cellular process; chromatin organization
SSR3	TRANSLOCON-ASSOCIATED PROTEIN SUBUNIT GAMMA	Viral mRNA translation; generic transcription pathway
SSR3	SOMATOSTATIN RECEPTOR TYPE 3	Cell communication; sensory perception
TAF15	TATA-BINDING PROTEIN-ASSOCIATED FACTOR 2N	RNA splicing, via trans-esterification reactions; mRNA splicing, via spliceosome; RNA splicing, via trans-esterification reactions; neurological system process
TBL1XR1	F-BOX-LIKE/WD REPEAT-CONTAINING PROTEIN TBL1XR1	Transcription co-repressor activity; protein N-terminus
TCEB3	TRANSCRIPTION ELONGATION FACTOR B POLYPEPTIDE 3	Transcription from RNA polymerase II promoter
TGFA	PROTRANSFORMING GROWTH FACTOR ALPHA	Cell cycle; cell communication
THRA	THYROID HORMONE RECEPTOR ALPHA	Transcription from RNA polymerase II promoter; cellular process; regulation of transcription from RNA polymerase II promoter
TMOD3	TROPOMODULIN-3	Cellular process; cellular component morphogenesis; muscle organ development; cellular component organization
TOB2	PROTEIN TOB2	Vitamin D receptor binding
TOMM22	MITOCHONDRIAL IMPORT RECEPTOR SUBUNIT TOM22 HOMOLOG	Protein binding; protein trans-membrane transporter activity
TPI1	TRIOSEPHOSPHATE ISOMERASE	Glycolysis
USP2	UBIQUITIN CARBOXYL-TERMINAL HYDROLASE 2	RNA splicing, via trans-esterification reactions; mRNA splicing, via spliceosome; RNA splicing, trans-esterification reactions; proteolysis
WDR1	WD REPEAT-CONTAINING PROTEIN 1	Cellular component movement
YWHAE	14-3-3 PROTEIN EPSILON	Cell cycle
YWHAZ	14-3-3 PROTEIN ZETA/DELTA	Cell cycle; cell communication

Supplementary Table S8: Gene names and functions represented in the MGMT interactome

Gene ID	Gene name	Biological processes
ACSL4	LONG-CHAIN-FATTY-ACID--COA LIGASE 4	Immune system process; fatty acid metabolic process; lipid transport
ANXA7	ANNEXIN A7	Fatty acid metabolic process
APBA2	AMYLOID BETA A4 PRECURSOR PROTEIN-BINDING FAMILY A MEMBER 2	Intracellular protein transport
ASCL1	ACHAETE-SCUTE HOMOLOG 1	Transcription from RNA polymerase II promoter; neurological system process; ectoderm development; nervous system development; regulation of transcription from RNA polymerase II promoter
ATF1	CYCLIC AMP-DEPENDENT TRANSCRIPTION FACTOR ATF-1	Immune system process; transcription from RNA polymerase II promoter; cell communication; neurological system process; ectoderm development; nervous system development; response to stress; regulation of transcription from RNA polymerase II promoter
B3GNT2	UDP-GLCNAC:BETAGAL BETA-1,3-N-ACETYLGLUCOSAMINYLTRANSFERASE	Female gamete generation; lipid metabolic process; translation; protein glycosylation; cell communication; dorsal/ventral axis specification; dorsal/ventral axis specification
BCAN	BREVICAN CORE PROTEIN	Cellular process; cell adhesion; ectoderm development; mesoderm development; nervous system development
CCL18	C-C MOTIF CHEMOKINE 18	Immune response; cellular process; response to stimulus
CD55	COMPLEMENT DECAY-ACCELERATING FACTOR	Complement activation; proteolysis; cell communication; cell-cell adhesion; blood coagulation; lipid transport
CFH	COMPLEMENT FACTOR H-RELATED	Complement activation; proteolysis; cell communication; cell-cell adhesion; blood coagulation ;lipid transport
CLEC7A	C-TYPE LECTIN DOMAIN FAMILY 7 MEMBER A	B cell mediated immunity; natural killer cell activation; cellular process; response to stimulus
COL15A1	COLLAGEN ALPHA-1(XV) CHAIN	Macrophage activation; cell communication; cell-cell adhesion; blood circulation; ectoderm development; mesoderm development; cellular component morphogenesis; response to stimulus; intracellular protein transport; receptor-mediated endocytosis; regulation of liquid surface tension; cellular component organization
CYP1B1	CYTOCHROME P450 1B1	Respiratory electron transport chain; fatty acid metabolic process; steroid metabolic process
DCLK2	SERINE/THREONINE-PROTEIN KINASE DCLK2	Protein phosphorylation; cell communication; nervous system development

DCN	DECORIN	Cell communication; cell-cell adhesion; mesoderm development; skeletal system development
DPP6	DIPEPTIDYL AMINOPEPTIDASE-LIKE PROTEIN 6	Immune system process; protein acetylation; proteolysis; cell communication ;cellular defence response
GCHFR	GTP CYCLOHYDROLASE 1 FEEDBACK REGULATORY PROTEIN	Enzyme inhibitor activity; GTP-dependent protein binding
GMFG	GLIA MATURATION FACTOR GAMMA	Metabolic process; ectoderm development; nervous system development; regulation of catalytic activity
GSTO1	GLUTATHIONE S-TRANSFERASE OMEGA-1	Immune system process; translation; cell communication; response to toxic substance; regulation of translation
HPRT1	HYPOXANTHINE-GUANINE PHOSPHORIBOSYLTRANSFERASE	Phosphate-containing compound metabolic process; nitrogen compound metabolic process; biosynthetic process; purine nucleobase metabolic process; cellular process
HTATIP2	OXIDOREDUCTASE HTATIP2	Oxidoreductase activity; nuclear binding
LUM	LUMICAN	Cellular process; cell adhesion; visual perception; sensory perception
LYN	TYROSINE-PROTEIN KINASE LYN	Apoptotic process; protein phosphorylation; cell-cell signalling; cell proliferation; cell adhesion; apoptotic process; cell differentiation
LYZ	LYSOZYME C	Lysozyme activity
MAPK13	MITOGEN-ACTIVATED PROTEIN KINASE 13	Immune system process; protein phosphorylation; cellular component movement; mitosis; cell communication; response to stress
MOB1A	MOB KINASE ACTIVATOR 1A	Metabolic process; cytokinesis; mitosis; regulation of catalytic activity
MST4	SERINE/THREONINE-PROTEIN KINASE MST4	Protein kinase activity; identical protein binding
NCAM1	NEURAL CELL ADHESION MOLECULE 1	Immune system process; induction of apoptosis; cellular protein modification process; cell cycle; cell-cell signalling; cell-cell adhesion; muscle contraction; neurological system process; ectoderm development; mesoderm development; induction of apoptosis; angiogenesis; nervous system development; muscle organ development
NOTCH1	NEUROGENIC LOCUS NOTCH HOMOLOG PROTEIN 1	Immune system process; transcription from RNA polymerase II promoter; cell-cell signalling; ectoderm development; nervous system development; regulation of transcription from RNA polymerase II promoter
NQO1	NAD(P)H DEHYDROGENASE [QUINONE] 1	NAD(P)H dehydrogenase (quinone) activity; cytochrome-b5 reductase activity, acting on NAD(P)H
OSTF1	OSTEOCLAST-STIMULATING FACTOR 1	SH3 binding domain
POMT1	PROTEIN O-MANNOSYL-TRANSFERASE 1	Translation; protein glycosylation
PTER	PHOSPHOTRIESTERASE-RELATED	Metabolic process

	PROTEIN	
SARM1	STERILE ALPHA AND TIR MOTIF-CONTAINING PROTEIN 1	Innate immune response
SIGIRR	SINGLE IG IL-1-RELATED RECEPTOR	Cellular process
SMARCA4	TRANSCRIPTION ACTIVATOR BRG1	DNA repair; DNA recombination; transcription from RNA polymerase II promoter; cellular process; regulation of transcription from RNA polymerase II promoter; chromatin organization
SOX11	TRANSCRIPTION FACTOR SOX-11	Transcription from RNA polymerase II promoter; regulation of transcription from RNA polymerase II promoter
SOX2	TRANSCRIPTION FACTOR SOX-2	Transcription from RNA polymerase II promoter; regulation of transcription from RNA polymerase II promoter
SPINT2	KUNITZ-TYPE PROTEASE INHIBITOR 2	Immune system process; proteolysis; blood coagulation; regulation of catalytic activity
STAT4	SIGNAL TRANSDUCER AND ACTIVATOR OF TRANSCRIPTION 4	Immune response; apoptotic process; transcription from RNA polymerase II promoter; cell communication; mesoderm development; apoptotic process; haemopoiesis; cellular defence response; regulation of transcription from RNA polymerase II promoter; negative regulation of apoptotic process
TANC2	PROTEIN TANC2	Protein phosphorylation
TCF3	TRANSCRIPTION FACTOR E2-ALPHA	Female gamete generation; transcription from RNA polymerase II promoter; ectoderm development; sex determination; nervous system development; regulation of transcription from RNA polymerase II promoter
TRIM9	E3 UBIQUITIN-PROTEIN LIGASE	Cellular protein modification process; neurotransmitter secretion; synaptic vesicle exocytosis
ZFAND1	AN1-TYPE ZINC FINGER PROTEIN 1	Zinc ion binding

Supplementary Table S9: Gene names and functions represented in the PTEN interactome

Gene ID	Gene name	Biological processes
ACP5	TARTRATE-RESISTANT ACID PHOSPHATASE TYPE 5	Phosphate-containing compound metabolic process; protein phosphorylation; proteolysis; cation transport
ADAM17	DISINTEGRIN AND METALLOPROTEINASE DOMAIN-CONTAINING PROTEIN 17	Integrin binding; metalloendopeptidase activity
ANP32A	ACIDIC LEUCINE-RICH NUCLEAR PHOSPHOPROTEIN 32 FAMILY MEMBER A-RELATED	Metabolic process; regulation of catalytic activity
BMS1	RIBOSOME BIOGENESIS PROTEIN BMS1 HOMOLOG	Nucleobase-containing compound metabolic process
C3orf18	CHROMOSOME 3 OPEN READING FRAME 18	Unknown
CDH1	CADHERIN-1	Cellular process; visual perception; sensory perception of sound; mesoderm development; nervous system development; heart development; muscle organ development
CDKL3	CYCLIN-DEPENDENT KINASE-LIKE 3	Glycogen metabolic process; protein phosphorylation; mitosis; cell communication; segment specification; segment specification; ectoderm development; mesoderm development; embryo development
CEP55	CENTROSOMAL PROTEIN OF 55 KDA	Mitotic exit; cytokinesis
CLDN4	CLAUDIN-4	Cellular process
COL15A1	COLLAGEN ALPHA-1(XV) CHAIN	Macrophage activation; cell communication; cell-cell adhesion; blood circulation; ectoderm development; mesoderm development; cellular component morphogenesis; response to stimulus; intracellular protein transport; receptor-mediated endocytosis; regulation of liquid surface tension; cellular component organization
CSTF2T	CLEAVAGE STIMULATION FACTOR SUBUNIT 2 TAU	RNA splicing, via trans-esterification reactions; mRNA splicing, via spliceosome; RNA splicing, via trans-esterification reactions

	VARIANT	
DLG3	DISKS LARGE HOMOLOG 3	Neuron-neuron synaptic transmission; cell adhesion; neurological system process; ectoderm development; cellular component morphogenesis; nervous system development; cellular component organization
DSC2	DESMOCOLLIN-2 (PTHR24025:SF0)	Cellular process
DSP	DESMOPLAKIN	Cellular component movement; cellular component morphogenesis; cellular component organization
EIF2S3	EUKARYOTIC TRANSLATION INITIATION FACTOR 2 SUBUNIT 3	Translation; regulation of translation
ELF3	ETS-RELATED TRANSCRIPTION FACTOR ELF-3	B cell mediated immunity; macrophage activation; transcription from RNA polymerase II promoter; cell cycle; cell communication; endoderm development; mesoderm development; haemopoiesis; cellular defence response; regulation of transcription from RNA polymerase II promoter
EML4	ECHINODERM MICROTUBULE-ASSOCIATED PROTEIN-LIKE 4	Microtubule assembly
EPCAM	EPITHELIAL CELL ADHESION MOLECULE	Cell communication
FAM178A	PROTEIN FAM178A	Unknown
FGD6	FYVE, RHOGEF AND PH DOMAIN-CONTAINING PROTEIN 6	Metabolic process; mesoderm development; skeletal system development; regulation of catalytic activity
FXYD3	FXYD DOMAIN-CONTAINING ION TRANSPORT REGULATOR 3	Cellular process; ion transport
GDI2	RAB GDP DISSOCIATION INHIBITOR BETA	Metabolic process; synaptic transmission; neurotransmitter secretion; intracellular protein transport; vesicle-mediated transport; regulation of catalytic activity
GFAP	GLIAL FIBRILLARY ACIDIC PROTEIN	Cellular process; cellular component morphogenesis; cellular component organization
GGA2	ADP-RIBOSYLATION FACTOR-BINDING	Metabolic process; cell communication; lysosomal transport; protein targeting; endocytosis; regulation of catalytic activity

	PROTEIN GGA2	
GM2A	GANGLIOSIDE GM2 ACTIVATOR	Protein lipidation
GOLPH3	GOLGI PHOSPHOPROTEIN 3	Lipid binding; protein binding
GTPBP4	NUCLEOLAR GTP-BINDING PROTEIN 1	Translation
HHEX	HEMATOPOIETICALLY-EXPRESSED HOMEOBOX PROTEIN HHEX	Transcription from RNA polymerase II promoter; regulation of transcription from RNA polymerase II promoter
HIF1AN	HYPOXIA-INDUCIBLE FACTOR 1-ALPHA INHIBITOR	Oxidoreductase activity; acting on single donors with incorporation of molecular oxygen; incorporation of two atoms of oxygen and protein homodimerization activity
HNRNPA3	HETEROGENEOUS NUCLEAR RIBONUCLEOPROTEIN A3	DNA replication; RNA splicing, via trans-esterification reactions; mRNA splicing, via spliceosome; mRNA polyadenylation; RNA splicing, via trans-esterification reactions; rRNA metabolic process; protein metabolic process; cell cycle; neurological system process; ectoderm development; nervous system development
HNRNPH1	HETEROGENEOUS NUCLEAR RIBONUCLEOPROTEIN H	mRNA splicing, via spliceosome
HSP90AB1	HEAT SHOCK PROTEIN HSP 90-BETA	Immune system process; protein folding; response to stress
JUP	JUNCTION PLAKOGLOBIN	Female gamete generation; nitrogen compound metabolic process; biosynthetic process; transcription from RNA polymerase II promoter; cell-cell signalling; cell adhesion; pattern specification process; pattern specification process; cellular component morphogenesis; embryo development; cell differentiation; heart development; response to stimulus; protein localization; regulation of transcription from RNA polymerase II promoter; cytoskeleton organization
KLF11	KRUEPPEL-LIKE FACTOR 11	B cell mediated immunity; transcription from RNA polymerase II promoter; anterior/posterior axis specification; anterior/posterior axis specification; mesoderm development; system development; response to stimulus; regulation of transcription from RNA polymerase II promoter
KLF5	KRUEPPEL-LIKE FACTOR 5	B cell mediated immunity; transcription from RNA polymerase II promoter; anterior/posterior axis specification; anterior/posterior axis specification; mesoderm development; system development; response to stimulus; regulation of

		transcription from RNA polymerase II promoter
KPNA6	IMPORTIN SUBUNIT ALPHA-7	Protein targeting; nuclear transport
KRT19	KERATIN, TYPE I CYTOSKELETAL 19	Cellular process; cellular component morphogenesis; cellular component organization
KRT8	KERATIN, TYPE II CYTOSKELETAL 8	Cellular process; cellular component morphogenesis; cellular component organization
LAMB3	LAMININ SUBUNIT BETA-3	Transcription from RNA polymerase II promoter; cell communication; cell-matrix adhesion; cell-cell adhesion; neurological system process; ectoderm development; nervous system development; regulation of transcription from RNA polymerase II promoter
LAMP1	LYSOSOME-ASSOCIATED MEMBRANE GLYCOPROTEIN 1	Proteolysis; lysosomal transport; intracellular protein transport
LARP4B	LA-RELATED PROTEIN 4B	tRNA metabolic process
LSR	LIPOLYSIS-STIMULATED LIPOPROTEIN RECEPTOR	Probable role in the clearance of triglyceride-rich lipoprotein from blood; binds chylomicrons, LDL and VLDL in presence of free fatty acids and allows their subsequent uptake in the cells
LTA4H	LEUKOTRIENE A-4 HYDROLASE	Fatty acid biosynthetic process; proteolysis
MAN1A1	MANNOSYL-OLIGOSACCHARIDE 1,2-ALPHA-MANNOSIDASE IA	Protein folding; proteolysis
MBTPS1	MEMBRANE-BOUND TRANSCRIPTION FACTOR SITE-1 PROTEASE	Protein metabolic process
MCM10	PROTEIN MCM10 HOMOLOG	DNA binding; metal ion binding
MLPH	MELANOPHILIN	Metabolic process; cellular process; cellular component morphogenesis; intracellular protein transport; vesicle-mediated transport; regulation of catalytic activity; cellular component organization
MYO1B	UNCONVENTIONAL MYOSIN-IB	Metabolic process; cytokinesis; cellular component movement; mitosis; cell communication; muscle contraction; sensory perception of sound; sensory

		perception; mesoderm development; cellular component morphogenesis; muscle organ development; intracellular protein transport; vesicle-mediated transport; regulation of catalytic activity; cellular component organization
NET1	NEUROEPITHELIAL CELL-TRANSFORMING GENE 1 PROTEIN	Apoptotic process; cellular amino acid metabolic process; cell cycle; cell communication; cell adhesion; apoptotic process; amino acid transport; intracellular protein transport; phagocytosis; regulation of catalytic activity
NET1	NETRIN-1	Cell communication; cell-matrix adhesion; cell-cell adhesion; neurological system process; ectoderm development; nervous system development
NIPBL	NIPPED-B-LIKE PROTEIN	DNA repair; cellular process; chromatin organization
PPP2R2D	SERINE/THREONINE-PROTEIN PHOSPHATASE 2A 55 KDA REGULATORY SUBUNIT B DELTA ISOFORM	Immune system process; protein phosphorylation; response to stress
PPRC1	PEROXISOME PROLIFERATOR-ACTIVATED RECEPTOR GAMMA COACTIVATOR-RELATED PROTEIN 1	Transcription from RNA polymerase II promoter; lipid metabolic process; regulation of transcription from RNA polymerase II promoter
PRKCI	PROTEIN KINASE C IOTA TYPE	Protein phosphorylation; cell communication
PRPF4B	SERINE/THREONINE-PROTEIN KINASE PRP4 HOMOLOG	Glycogen metabolic process; protein phosphorylation; mitosis; cell communication
RAB11FIP1	RAB11 FAMILY-INTERACTING PROTEIN 1 (PTHR15746:SF22)	Probably role in regulating Rab GTPases
RAB25	RAS-RELATED PROTEIN RAB-25	GTP binding
REEP2	RECEPTOR EXPRESSION-ENHANCING PROTEIN 2	Carbohydrate metabolic process; carbohydrate transport
RIPK4	RECEPTOR-	Protein serine/threonine kinase activity; ATP binding

	INTERACTING SERINE/THREONINE-PROTEIN KINASE 4	
RMND5A	PROTEIN RMD5 HOMOLOG A	Unknown
SEC23IP	SEC23-INTERACTING PROTEIN	Metabolic process; intracellular protein transport; vesicle-mediated transport
SEL1L3	PROTEIN SEL-1 HOMOLOG 3	Metabolic process; regulation of catalytic activity
SGPL1	SPHINGOSINE-1-PHOSPHATE LYASE 1	Cellular amino acid metabolic process
SMARCC1	SWI/SNF COMPLEX SUBUNIT SMARCC1	Transcription from RNA polymerase II promoter; regulation of transcription from RNA polymerase II promoter
SMC3	STRUCTURAL MAINTENANCE OF CHROMOSOMES PROTEIN 3	DNA replication; DNA repair; mitosis; meiosis; chromosome segregation; chromatin organization
SPINT1	KUNITZ-TYPE PROTEASE INHIBITOR 1	Immune system process; proteolysis; blood coagulation; regulation of catalytic activity
ST14	SUPPRESSOR OF TUMORIGENICITY 14 PROTEIN	Fertilization; immune system process; apoptotic process; lipid metabolic process; blood circulation; mesoderm development; apoptotic process; angiogenesis; blood coagulation; lipid transport; regulation of catalytic activity
SVIL	SUPERVILLIN	Cellular process; cellular component morphogenesis; cellular component organization
TACSTD2	TUMOR-ASSOCIATED CALCIUM SIGNAL TRANSDUCER 2	Cell communication
TBC1D4	TBC1 DOMAIN FAMILY MEMBER 4	Metabolic process; cellular process; cellular component morphogenesis; intracellular protein transport; exocytosis ;regulation of catalytic activity; cellular component organization
TBL1XR1	F-BOX-LIKE/WD REPEAT-CONTAINING PROTEIN TBL1XR1	Transcription co-repressor activity; protein N-terminus binding
TMEM30B	CELL CYCLE CONTROL PROTEIN 50B	Protein binding
TMOD3	TROPOMODULIN-3	Cellular process; cellular component morphogenesis; muscle organ development; cellular component organization

TMPO	LAMINA-ASSOCIATED POLYPEPTIDE 2, ISOFORM ALPHA	Immune system process; cellular defence response
TNFAIP8	TUMOR NECROSIS FACTOR ALPHA-INDUCED PROTEIN 8	Immune system process; response to stress
TNKS2	TANKYRASE-2	Immune system process; DNA replication; transcription, DNA-dependent; protein ADP-ribosylation; protein ADP-ribosylation; cell cycle; response to stress
TSPAN1	TETRASPANIN-1	Gamete generation; immune system process; cell communication; cell-cell adhesion; neurological system process; blood coagulation
VANGL1	VANG-LIKE PROTEIN 1	Protein binding

Supplementary Table S10: Clinicopathological associations of *APE1* gene expression in the Nottingham cohort

Variable	*APE1* Expression		X^2
	Low	High	*Adjusted p value*
	N= 25	N= 35	
**Demographics**			
Age			
<59 years	14 (56.0)	25 (71.4)	0.217
≥59 years	11 (44.0)	10 (28.6)	
Gender			
Male	16 (64.0)	23 (65.7)	0.891
Female	9 (36.0)	12 (34.3)	
Previous glioma diagnosis			
No	18 (72.0)	25 (71.4)	0.961
Yes	7 (28.0)	10 (28.6)	
Grade			
3	5 (20.0	14 (40.0)	0.101
4	20 (80.0)	21 (60.0)	
**Treatment given**			
Radiotherapy			
No	0 (0.0)	2 (6.2)	0.666*
Yes	21 (100.0)	30 (93.8)	

Chemotherapy			
No	7 (33.3)	9 (27.3)	0.634
Yes	14 (66.7)	24 (72.7)	
**DNA repair gene expression**			
NBN expression			
Low	17 (68.0)	20 (57.1)	0.394
High	8 (32.0)	15 (42.9)	
Nuclear PTEN expression			
Low	24 (96.0)	25 (71.4)	**0.037***
High	1 (4.0)	10 (28.6)	
Cytoplasmic PTEN expression			
Low	13 (52.0)	14 (40.0)	0.357
High	12 (48.0)	21 (60.0)	
PMS2 expression			
Low	10 (40.0)	4 (11.4)	**0.010**
High	15 (0.60)	31 (88.6)	

* Yates' continuity correction. Significant p values (≤0.05) are shown in bold.

Supplementary Table 11: Clinicopathological associations of *NBN* gene expression in the Nottingham Cohort

Variable	*NBN* Expression Low N=37	*NBN* Expression High N=24	X^2 *Adjusted p value*
**Demographics**			
Age			
<59 years	24 (64.9)	16 (66.7)	0.885
≥59 years	13 (35.1)	8 (33.3)	
Gender			
Male	23 (62.2)	16 (66.7)	0.720
Female	14 (37.8)	8 (33.3)	
Previous glioma diagnosis			
No	28 (75.7)	15 (62.5)	0.270
Yes	9 (24.3)	9 (37.5)	
Grade			
3	9 (24.3)	11 (45.8)	0.080
4	28 (75.7)	13 (54.2)	
**Treatment given**			
Radiotherapy			
No	1 (3.1)	1 (4.5)	1.000*
Yes	31 (96.9)	21 (95.5)	

Chemotherapy			
No	9 (27.3)	7 (31.8)	0.716
Yes	24 (72.7)	15 (68.2)	
**DNA repair gene expression**			
APE1 expression			
Low	17 (45.9)	8 (34.8)	0.394
High	20 (54.1)	15 (65.2)	
Nuclear PTEN expression			
Low	33 (89.2)	17 (70.8)	0.139*
High	4 (10.8)	7 (29.2)	
Cytoplasmic PTEN expression			
Low	17 (45.9)	10 (41.7)	0.948
High	20 (54.1)	14 (58.3)	
PMS2 expression			
Low	9 (24.3)	5 (20.8)	0.996*
High	28 (75.7)	19 (79.2)	

* Yates' continuity correction. Significant p values (≤0.05) are shown in bold.

Supplementary Table S12: Clinicopathological associations of nuclear *PMS2* gene expression in the Nottingham Cohort

Variable	*PMS2* Expression Low N=14	High N=47	X^2 *Adjusted p value*
**Demographics**			
Age			
<59 years	8 (57.1)	32 (68.1)	0.663*
≥59 years	6 (42.9)	15 (31.9)	
Gender			
Male	10 (71.4)	29 (61.7)	0.728*
Female	4 (28.6)	18 (38.3)	
Previous glioma diagnosis			
No	12 (85.7)	31 (66.0)	0.276*
Yes	2 (14.3)	16 (34.0)	
Grade			
3	3 (21.4)	17 (36.2)	0.480*
4	11 (78.6)	30 (63.8)	
**Treatment given**			
Radiotherapy			
No	0 (0.0)	2 (4.7)	1.00*
Yes	11 (100.0)	41 (95.3)	

Chemotherapy			
No	3 (27.3)	13 (29.5)	1.00*
Yes	8 (72.7)	31 (70.5)	
**DNA repair gene expression**			
APE1 expression			
Low	10 (71.4)	15 (32.6)	**0.010**
High	4 (28.6)	31 (67.4)	
NBN expression			
Low	9 (64.3)	28 (59.6)	0.996*
High	5 (35.7)	19 (18.5)	
Nuclear PTEN expression			
Low	13 (92.9)	37 (38.5)	0.417*
High	1 (7.1)	10 (21.3)	
Cytoplasmic PTEN expression			
Low	8 (57.1)	19 (40.4)	0.269
High	6 (42.9)	28 (59.6)	

* Yates' continuity correction. Significant p values (≤0.05) are shown in bold.

Supplementary Table S13: Clinicopathological associations of nuclear *PTEN* gene expression in the Nottingham Cohort.

Variable	Nuclear *PTEN* Expression		X^2
	**Low**	**High**	***Adjusted p value***
	**N=50**	**N=11**	
**Demographics**			
Age			
<59 years	31 (62.0)	9 (81.8)	0.367*
≥59 years	19 (38.0)	2 (18.2)	
Gender			
Male	31 (62.0)	8 (72.7)	0.746*
Female	19 (38.0)	3 (27.3)	
Previous glioma diagnosis			
No	35 (70.0)	8 (72.7)	1.000*
Yes	15 (30.0)	3 (27.3)	
Grade			
3	12 (24.0)	8 (72.7)	**0.006**
4	38 (76.0)	3 (27.3)	
**Treatment given**			
Radiotherapy			
No	1 (2.3)	1 (10.0)	0.810*
Yes	43 (97.7)	9 (90.0)	
Chemotherapy			
No	14 (31.1)	2 (20.0)	0.753
Yes	31 (68.9)	8 (80.0)	

**DNA repair gene expression**			
APE1 expression			
Low	24 (49.0)	1 (9.1)	**0.037***
High	25 (51.0	10 (90.9)	
NBN expression			
Low	33 (66.0)	4 (36.4)	0.139*
High	17 (34.0)	7 (63.6)	
Cytoplasmic PTEN expression			
Low	25 (50.0)	2 (18.2)	0.112*
High	25 (50.0)	9 (81.8)	
PMS expression			
Low	13 (26.0)	1 (9.1)	0.417*
High	37 (74.0)	10 (90.9)	

* Yates' continuity correction. Significant p values (≤0.05) are shown in bold.

Supplementary Table S14: Clinicopathological associations of cytoplasmic *PTEN* gene expression in the Nottingham Cohort

**Variable**	**Cytoplasmic *PTEN* Expression**		X^2
	**Low**	**High**	***Adjusted p value***
	**N=27**	**N=34**	
**Demographics**			
Age			
<59 years	16 (59.3)	24 (70.6)	0.355
≥59 years	11 (40.7)	10 (29.4)	
Gender			
Male	16 (59.3)	23 (67.6)	0.498
Female	11 (40.7)	11 (32.4)	
Previous glioma diagnosis			
No	22 (81.5)	21 (61.8)	0.094
Yes	5 (18.5)	13 (38.2)	
Grade			
3	5 (18.5)	15 (44.1)	**0.034**
4	22 (81.5)	19 (55.9)	
**Treatment given**			
Radiotherapy			
No	1 (4.2)	1 (3.3)	1.000*
Yes	23 (95.8)	29 (96.7)	
Chemotherapy			
No	7 (29.2)	9 (29.0)	0.991
Yes	17 (70.8)	22 (71.0)	

**DNA repair gene expression**			
APE1 expression			
Low	13 (48.1)	12 (36.4)	0.357
High	14 (51.9)	21 (63.6)	
NBN expression			
Low	17 (63.0)	20 (58.8)	0.742
High	10 (37.0)	14 (41.2)	
Nuclear PTEN expression			
Low	25 (92.6)	25 (73.5)	0.112*
High	2 (7.4)	9 (26.5)	
PMS2 expression			
Low	8 (29.6)	6 (17.6)	0.269
High	19 (70.4)	28 (82.4)	

* Yates' continuity correction. Significant p values (≤0.05) are shown in bold.

Supplementary Table S15: Baseline demographic data for the TCGA dataset (n=508)

Clinicopathological variable	Number (%)
Gender	
Male	308 (60.6%)
Female	200 (39.4)
Karnofsky performance status	
100	54 (10.6%)
80-90	231 (45.5%)
60-70	83 (16.3%)
40-50	11 (2.2%)
0-30	2 (0.4%)
Unknown	127 (25.0%)
Survival Status	
Alive	92 (18.1%)
Deceased	416 (81.9%)
Radiotherapy given	
Yes	373 (73.4%)
No	135 (26.6%)
Chemotherapy given	
Yes	351 (69.1%)
No	157 (30.9%)
Type of chemotherapy given:	
Temozolomide	294 (57.9%)
PCV[1]	26 (5.1%)
BCNU (Carmustine)	17 (3.3%)
Bevacizumab	73 (14.4%)
Targeted agent	39 (7.7%)
Gliadel wafer	30 (5.9%)
Other	99 (19.5)

[1] Procarbazine, lomustine and vincristine

Supplementary Table S16: List of 188 DNA repair genes

**DNA repair gene**	**Pathway**
CCNO	Base excision repair
MBD4	Base excision repair
MPG	Base excision repair
MUTYH (MYH)	Base excision repair
NEIL1	Base excision repair
NEIL2	Base excision repair
NEIL3	Base excision repair
NTHL1 (NTH1)	Base excision repair
OGG1	Base excision repair
SMUG1	Base excision repair
TDG	Base excision repair
UNG	Base excision repair
APEX1 (APE1)	Base excision repair and strand break joining factors
APEX2	Base excision repair and strand break joining factors
APLF (C2ORF13)	Base excision repair and strand break joining factors
LIG3	Base excision repair and strand break joining factors
PNKP	Base excision repair and strand break joining factors
XRCC1	Base excision repair and strand break joining factors
PARP1 (ADPRT)	Poly(ADP-ribose) polymerase (PARP) enzymes that bind to DNA
PARP2 (ADPRTL2)	Poly(ADP-ribose) polymerase (PARP) enzymes that bind to DNA
PARP3 (ADPRTL3)	Poly(ADP-ribose) polymerase (PARP) enzymes that bind to DNA
ALKBH2 (ABH2)	Direct reversal of damage
ALKBH3 (DEPC1)	Direct reversal of damage
MGMT	Direct reversal of damage
TDP1	Repair of DNA-topoisomerase crosslinks
TDP2 (TTRAP)	Repair of DNA-topoisomerase crosslinks
MLH1	Mismatch excision repair
MLH3	Mismatch excision repair

MSH2	Mismatch excision repair
MSH3	Mismatch excision repair
MSH4	Mismatch excision repair
MSH5	Mismatch excision repair
MSH6	Mismatch excision repair
PMS1	Mismatch excision repair
PMS2	Mismatch excision repair
PMS2L3	Mismatch excision repair
CCNH	Nucleotide excision repair
CDK7	Nucleotide excision repair
CETN2	Nucleotide excision repair
DDB1	Nucleotide excision repair
DDB2 (XPE)	Nucleotide excision repair
ERCC1	Nucleotide excision repair
ERCC2 (XPD)	Nucleotide excision repair
ERCC3 (XPB)	Nucleotide excision repair
ERCC4 (XPF)	Nucleotide excision repair
ERCC5 (XPG)	Nucleotide excision repair
ERCC6 (CSB)	Nucleotide excision repair
ERCC8 (CSA)	Nucleotide excision repair
GTF2H1	Nucleotide excision repair
GTF2H2	Nucleotide excision repair
GTF2H3	Nucleotide excision repair
GTF2H4	Nucleotide excision repair
GTF2H5 (TTDA)	Nucleotide excision repair
LIG1	Nucleotide excision repair
MMS19	Nucleotide excision repair
MNAT1	Nucleotide excision repair
RAD23A	Nucleotide excision repair
RAD23B	Nucleotide excision repair

RPA1	Nucleotide excision repair
RPA2	Nucleotide excision repair
RPA3	Nucleotide excision repair
SLK	Nucleotide excision repair
TFIIH	Nucleotide excision repair
UVSSA (KIAA1530)	Nucleotide excision repair
XAB2 (HCNP)	Nucleotide excision repair
XPA	Nucleotide excision repair
XPC	Nucleotide excision repair
BRCA1	Homologous recombination
DMC1	Homologous recombination
EME1 (MMS4L)	Homologous recombination
EME2	Homologous recombination
GEN1	Homologous recombination
GIYD1 (SLX1A)	Homologous recombination
GIYD2 (SLX1B)	Homologous recombination
MRE11A	Homologous recombination
MUS81	Homologous recombination
NBN (NBS1)	Homologous recombination
RAD21	Homologous recombination
RAD50	Homologous recombination
RAD51	Homologous recombination
RAD51B	Homologous recombination
RAD51D	Homologous recombination
RAD52	Homologous recombination
RAD54B	Homologous recombination
RAD54L	Homologous recombination
RBBP8 (CtIP)	Homologous recombination
SHFM1 (DSS1)	Homologous recombination
XRCC2	Homologous recombination

XRCC3	Homologous recombination
BRCA2 (FANCD1)	Fanconi anaemia
BRIP1 (FANCJ)	Fanconi anaemia
BTBD12 (SLX4) (FANCP)	Fanconi anaemia
FAAP20 (C1orf86)	Fanconi anaemia
FAAP24 (C19orf40)	Fanconi anaemia
FANCA	Fanconi anaemia
FANCB	Fanconi anaemia
FANCC	Fanconi anaemia
FANCD2	Fanconi anaemia
FANCE	Fanconi anaemia
FANCF	Fanconi anaemia
FANCG (XRCC9)	Fanconi anaemia
FANCI (KIAA1794)	Fanconi anaemia
FANCL	Fanconi anaemia
FANCM	Fanconi anaemia
PALB2 (FANCN)	Fanconi anaemia
RAD51C (FANCO)	Fanconi anaemia
DCLRE1C (Artemis)	Non-homologous end-joining
DUT	Non-homologous end-joining
LIG4	Non-homologous end-joining
NHEJ1 (XLF, Cernunnos)	Non-homologous end-joining
NUDT1 (MTH1)	Non-homologous end-joining
PRKDC	Non-homologous end-joining
RRM2B (p53R2)	Non-homologous end-joining
XRCC4	Non-homologous end-joining
XRCC5 (Ku80)	Non-homologous end-joining
XRCC6 (Ku70)	Non-homologous end-joining
MAD2L2 (REV7)	DNA polymerases (catalytic subunits)
PCNA	DNA polymerases (catalytic subunits)

POLB	DNA polymerases (catalytic subunits)
POLD1	DNA polymerases (catalytic subunits)
POLD3	DNA polymerases (catalytic subunits)
POLE	DNA polymerases (catalytic subunits)
POLG	DNA polymerases (catalytic subunits)
POLH	DNA polymerases (catalytic subunits)
POLI (RAD30B)	DNA polymerases (catalytic subunits)
POLK (DINB1)	DNA polymerases (catalytic subunits)
POLL	DNA polymerases (catalytic subunits)
POLM	DNA polymerases (catalytic subunits)
POLN (POL4P)	DNA polymerases (catalytic subunits)
POLQ	DNA polymerases (catalytic subunits)
REV1L (REV1)	DNA polymerases (catalytic subunits)
REV3L (POLZ)	DNA polymerases (catalytic subunits)
APTX (aprataxin)	Editing and processing nucleases
ENDOV	Editing and processing nucleases
EXO1 (HEX1)	Editing and processing nucleases
FAN1 (MTMR15)	Editing and processing nucleases
FEN1 (DNase IV)	Editing and processing nucleases
SPO11	Editing and processing nucleases
TREX1 (DNase III)	Editing and processing nucleases
TREX2	Editing and processing nucleases
HLTF (SMARCA3)	Ubiquitination and modification
RAD18	Ubiquitination and modification
RNF168	Ubiquitination and modification
RNF4	Ubiquitination and modification
RNF8	Ubiquitination and modification
SHPRH	Ubiquitination and modification
SPRTN (c1orf124)	Ubiquitination and modification
UBE2A (RAD6A)	Ubiquitination and modification

UBE2B (RAD6B)	Ubiquitination and modification
UBE2N (UBC13)	Ubiquitination and modification
UBE2V2 (MMS2)	Ubiquitination and modification
CHAF1A (CAF1)	Chromatin Structure and Modification
H2AFX (H2AX)	Chromatin Structure and Modification
SETMAR (METNASE)	Chromatin Structure and Modification
ATM	Genes defective in diseases associated with sensitivity to DNA damaging agents
BLM	Genes defective in diseases associated with sensitivity to DNA damaging agents
RECQL4	Genes defective in diseases associated with sensitivity to DNA damaging agents
TTDN1 (C7orf11)	Genes defective in diseases associated with sensitivity to DNA damaging agents
WRN	Genes defective in diseases associated with sensitivity to DNA damaging agents
DCLRE1A (SNM1)	Other identified genes with known or suspected DNA repair function
DCLRE1B (SNM1B)	Other identified genes with known or suspected DNA repair function
HELQ (HEL308)	Other identified genes with known or suspected DNA repair function
OBFC2B (SSB1)	Other identified genes with known or suspected DNA repair function
PRPF19 (PSO4)	Other identified genes with known or suspected DNA repair function
RDM1 (RAD52B)	Other identified genes with known or suspected DNA repair function
RECQL (RECQ1)	Other identified genes with known or suspected DNA repair function
RECQL5	Other identified genes with known or suspected DNA repair function
RPA4	Other identified genes with known or suspected DNA repair function
ATR	Other conserved DNA damage response genes
ATRIP	Other conserved DNA damage response genes
CHEK1	Other conserved DNA damage response genes
CHEK2	Other conserved DNA damage response genes
CLK2	Other conserved DNA damage response genes
HUS1	Other conserved DNA damage response genes
MDC1	Other conserved DNA damage response genes
PER1	Other conserved DNA damage response genes
RAD1	Other conserved DNA damage response genes
RAD17 (RAD24)	Other conserved DNA damage response genes

RAD9A	Other conserved DNA damage response genes
RFC1	Other conserved DNA damage response genes
RIF1	Other conserved DNA damage response genes
TOP3A	Other conserved DNA damage response genes
TOP3B	Other conserved DNA damage response genes
TOPBP1	Other conserved DNA damage response genes
TP53	Other conserved DNA damage response genes
TP53BP1 (53BP1)	Other conserved DNA damage response genes
XRCC6BP1	Other conserved DNA damage response genes

Supplementary Table S17: Baseline demographic data for Nottingham Cohort (n=61)

Clinicopathological variable	Number (%)
Gender	
Male	39 (63.9)
Female	22 (36.1)
Survival Status	
Alive	19 (31.1)
Deceased	42 (68.9)
Previous diagnosis of glioma	
None	43 (70.5)
Grade 2	8 (13.1)
Grade 3	1 (1.6)
Grade 3-4	1 (1.6)
Grade 4	1 (1.6)
Low grade on imaging	7 (11.5)
Trial pathology	
Grade 3 (n=20)	
Anaplastic astrocytoma[1]	12 (19.7)
Anaplastic oligoastrocytoma[2]	4 (6.6)
Anaplastic oligodenodroglioma	4 (6.6)
Grade 4 (n=41)	
Glioblastoma	41 (67.2)
Surgery	
Biopsy	12 (19.7)
Debulking	49 (80.3)
Radiotherapy given	
Yes	52 (85.2)
No	2 (3.3)
Unknown	7 (11.5)
Chemotherapy given	
Yes	16 (26.2)
No	39 (63.9)
Unknown	6 (9.8)

[1]One anaplastic oligoastrocytoma classified as grade2-3, [2]Two anaplastic astrocytoma classified as grade 2-3

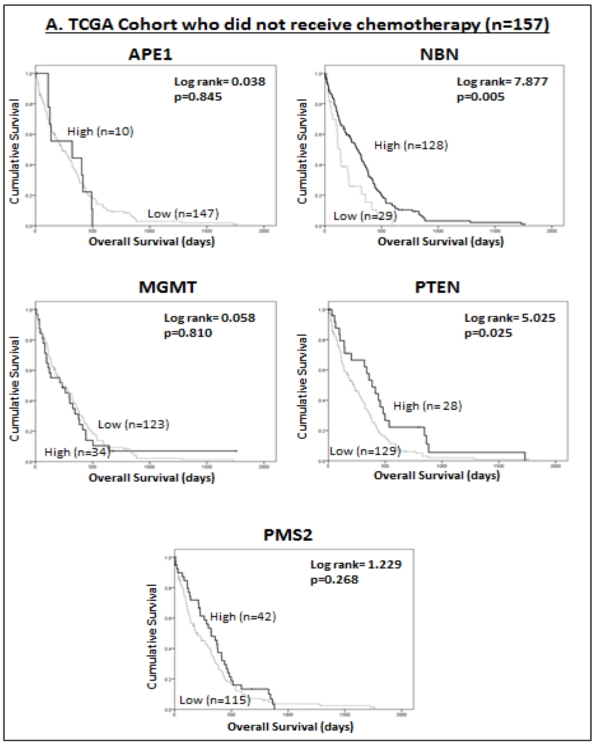

A. TCGA Cohort who did not receive chemotherapy (n=157)
APE1
Log rank= 0.038
p=0.845
High (n=10)
Low (n=147)
NBN
Log rank= 7.877
p=0.005
High (n=128)
Low (n=29)
MGMT
Log rank= 0.058
p=0.810
Low (n=123)
High (n=34)
PTEN
Log rank= 5.025
p=0.025
High (n= 28)
Low (n=129)
PMS2
Log rank= 1.229
p=0.268
High (n=42)
Low (n=115)
Cumulative Survival
Overall Survival (days)

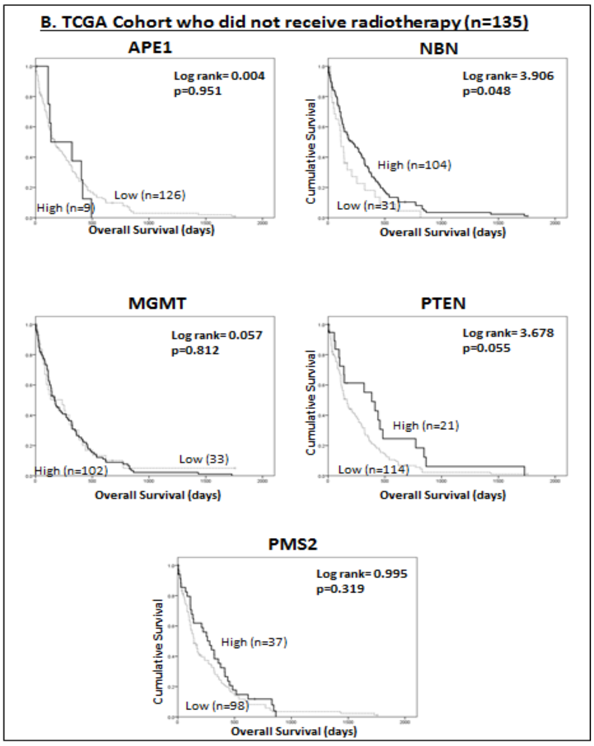

B. TCGA Cohort who did not receive radiotherapy (n=135)
APE1
Log rank= 0.004
p=0.951
Low (n=126)
High (n=9)
NBN
Log rank= 3.906
p=0.048
High (n=104)
Low (n=31)
MGMT
Log rank= 0.057
p=0.812
Low (33)
High (n=102)
PTEN
Log rank= 3.678
p=0.055
High (n=21)
Low (n=114)
PMS2
Log rank= 0.995
p=0.319
High (n=37)
Low (n=98)
Cumulative Survival
Overall Survival (days)

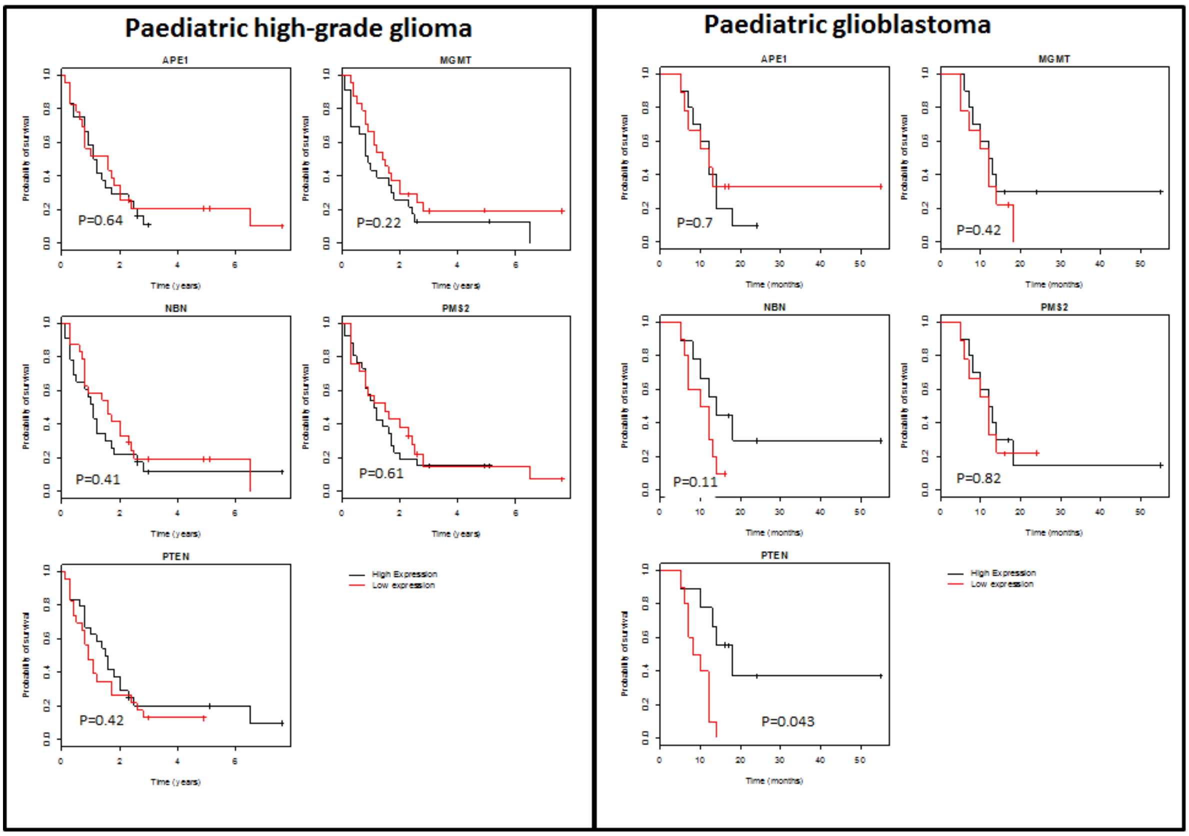
Paediatric high-grade glioma
APE1
P=0.64
MGMT
P=0.22
NBN
P=0.41
PMS2
P=0.61
PTEN
P=0.42
Probability of survival
Time (years)
High Expression
Low expression
Paediatric glioblastoma
APE1
P=0.7
MGMT
P=0.42
NBN
P=0.11
PMS2
P=0.82
PTEN
P=0.043
Probability of survival
Time (months)
High Expression
Low expression

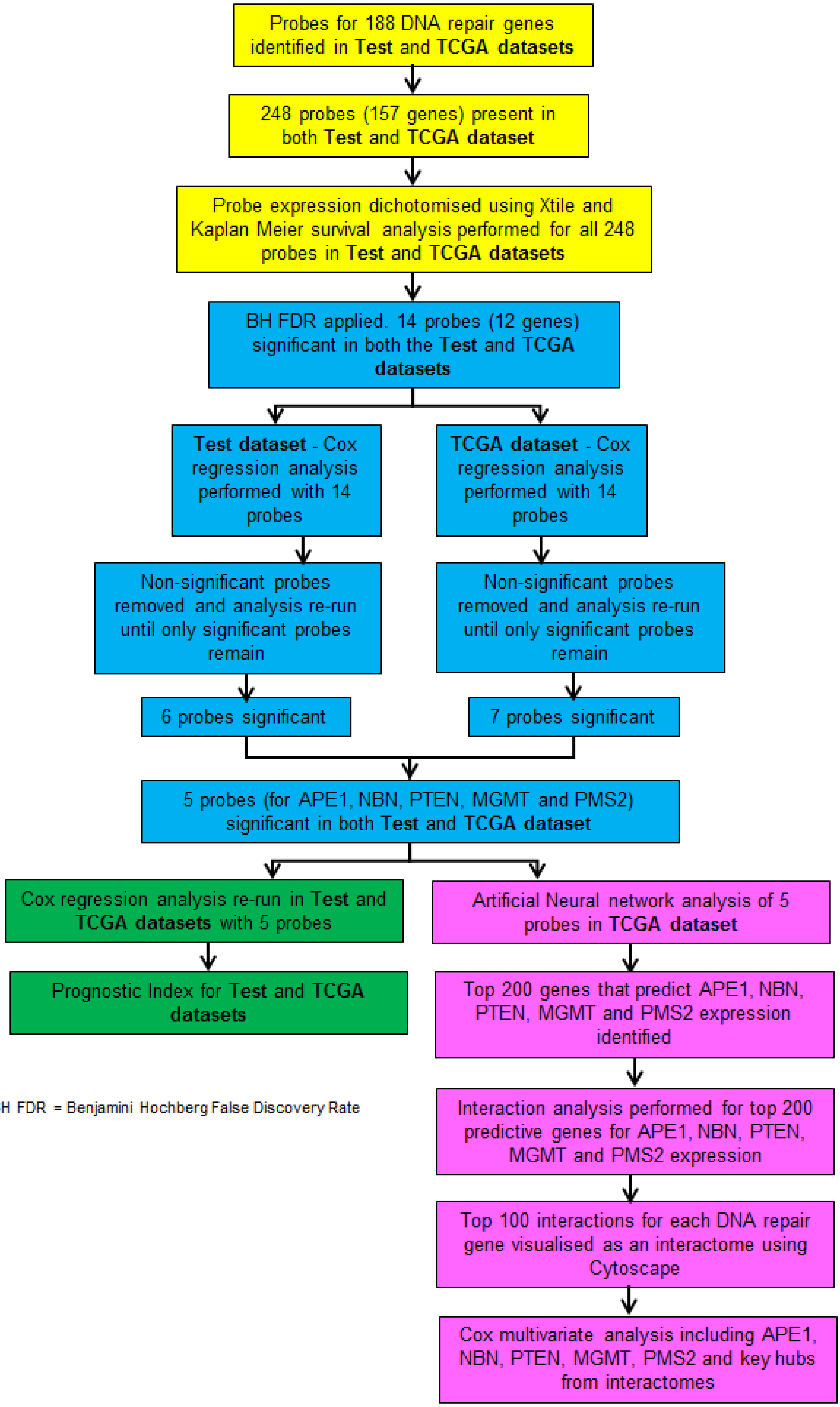

BH FDR = Benjamini Hochberg False Discovery Rate
